# Review of Evolocumab for the Reduction of LDL Cholesterol and Secondary Prevention of Atherosclerotic Cardiovascular Disease

**DOI:** 10.31083/j.rcm2505190

**Published:** 2024-05-23

**Authors:** Lawrence A. Leiter, Robert A. Hegele, Vivien Brown, Jean Bergeron, Erin S. Mackinnon, G. B. John Mancini

**Affiliations:** ^1^Li Ka Shing Knowledge Institute, St. Michael’s Hospital, University of Toronto, Toronto, ON M5S 1A8, Canada; ^2^Departments of Medicine and Biochemistry, Schulich School of Medicine and Dentistry, Western University, London, ON N6A 5C1, Canada; ^3^Department of Family and Community Medicine, Temerty Faculty of Medicine, University of Toronto, Toronto, ON M5G 1V7, Canada; ^4^Departments of Laboratory Medicine and Medicine, Centre Hospitalier Universitaire de Québec, Université Laval, Québec, QC G1V 4G2, Canada; ^5^Amgen Canada Inc., ON L5N 0A4, Canada; ^6^Centre for Cardiovascular Innovation, Division of Cardiology, University of British Columbia, Vancouver, BC V5Z 1M9, Canada

**Keywords:** evolocumab, proprotein convertase subtilisin-kexin type 9 (PCSK9) inhibitor, lipid-lowering therapy, low-density lipoprotein cholesterol (LDL-C), atherosclerotic cardiovascular disease (ASCVD), cardiovascular outcomes

## Abstract

Elevated low-density lipoprotein cholesterol (LDL-C) is a major causal factor for atherosclerotic cardiovascular disease (ASCVD), the leading cause of mortality worldwide. Statins are the recommended first-line lipid-lowering therapy (LLT) for patients with primary hypercholesterolemia and established ASCVD, with LLT intensification recommended in the substantial proportion of patients who do not achieve levels below guideline-recommended LDL-C thresholds with statin treatment alone. The proprotein convertase subtilisin/kexin type 9 inhibitor monoclonal antibody evolocumab has demonstrated significant LDL-C reductions of >60% in the clinical trial and open-label extension settings, with LDL-C reductions observed early post-evolocumab initiation and maintained long term, during up to 8.4 years of follow-up. Evolocumab therapy, when added to a statin, also conferred a significant reduction in major cardiovascular (CV) events, including a 20% reduction in the composite of CV death, myocardial infarction (MI), or stroke. The absolute benefits were enhanced among various patient types at high and very high risk for secondary ASCVD (e.g., with recent MI, multiple events or peripheral artery disease). Importantly, evolocumab treatment resulted in incremental CV risk reductions during the extended follow-up, including a 23% reduction in CV mortality and no apparent LDL-C level below which there is no further CV risk reduction. Hence, the evolocumab clinical data support the need for early and significant LDL-C lowering, especially in vulnerable ASCVD patients, in order to derive the greatest benefit in the long term. Importantly, evolocumab had no impact on any treatment emergent adverse events apart from a small increase in local injection site reactions. A growing body of real-world evidence (RWE) for evolocumab in heterogeneous populations is consistent with the trial data, including robust LDL-C reductions below guideline-recommended thresholds, a favourable safety profile even at the lowest levels of LDL-C achieved, and a high treatment persistence rate of >90%. Altogether, this review highlights findings from 50 clinical trials and RWE studies in >51,000 patients treated with evolocumab, to demonstrate the potential of evolocumab to address the healthcare gap in LDL-C reduction and secondary prevention of ASCVD in a variety of high- and very high-risk patients.

## 1. Introduction

Chronically elevated low-density lipoprotein cholesterol (LDL-C) drives the development and manifestation of atherosclerotic cardiovascular disease (ASCVD), the leading cause of mortality worldwide, responsible for approximately one-third of all deaths [1]. For secondary ASCVD prevention, international guidelines recommend lipid-lowering therapy (LLT) to reduce LDL-C below target thresholds, generally either <1.8 mmol/L (<70 mg/dL) or <1.4 mmol/L (<55 mg/dL), depending on the country/region [2, 3, 4, 5]. Most guidelines also recommend an LDL-C reduction of ≥50% from baseline, particularly for primary ASCVD prevention in patients with familial hypercholesterolemia (FH) and LDL-C ≥2.5 mmol/L (≥97 mg/dL) [2, 3, 4]. Still, a high residual risk remains for recurrent major cardiovascular (CV) events in patients with ASCVD, including those with an acute myocardial infarction (MI) [6]. Hence, for such patients who experience a second vascular event within 2 years, European guidelines recommend a more intensive LDL-C goal of <1.0 mmol/L (<40.0 mg/dL) [3]. Importantly, the cardioprotective benefits of LLT compound over time, with an estimated 12% reduction in the risk of major CV events for every 1 mmol/L (40 mg/dL) reduction in LDL-C after 1 year, 20% after 3 years, 23% after 5 years and 29% after 7 years, thus underscoring the need for early, effective, and sustained intervention [7].

Statins are the recommended first-line LLT in patients who require lipid lowering [2, 3, 4, 5], yet real-world evidence (RWE) consistently reveals a substantial proportion of patients at high risk of or with established ASCVD do not achieve levels below guideline-recommended LDL-C thresholds despite maximally tolerated statin therapy [8, 9, 10]. In these patients, LLT intensification with non-statin therapies as recommended by international guidelines may include ezetimibe and/or proprotein convertase subtilisin/kexin type 9 inhibitor (PCSK9i) [2, 3, 4, 5], among other effective pharmacological options with varying mechanisms of action and efficacy now available (Table 1, Ref. [11, 12, 13, 14, 15, 16, 17, 18, 19]).

**Table 1.** **Lipid-lowering therapies approved in North America and/or Europe**.

LLT	Target	CV outcomes trial and treatment arms	Patient population	Mean LDL-C reduction shown in CV outcomes trial	Relative and absolute reduction in CV death, MI or stroke in the ASCVD population
Statins [11, 12, 13]	HMG-CoA reductase	TNT	N = 10,001 stable ASCVD (LDL-C <3.36 mmol/L [130 mg/dL])	•High intensity: ≥50%	RRR: 20.1% at 5 years
				•Moderate intensity: 30% to 50%	ARR: 2.2% at 5 years
		•Intensive therapy with atorvastatin 80 mg		•Low intensity: <30%	
		•Moderate regimen of atorvastatin 10 mg			
Ezetimibe [14]	NPC1L1	IMPROVE-IT	N = 18,144 patients hospitalized for ACS within past 10 days (LDL-C >1.29 mmol/L [50 mg/dL])	24%	RRR: 5.7% at 7 years
		•Simvastatin 40 mg plus ezetimibe 10 mg			ARR: 2.0% at 7 years
		•Placebo (simvastatin 40 mg)			
Bempedoic acid [15]	ATP-citrate lyase	CLEAR OUTCOMES	N = 13,970 statin-intolerant with or at high-risk of ASCVD	21.1%	RRR: 13.6% at 5 years
		•Bempedoic acid 180 mg			ARR: 1.3% at 5 years
		•Placebo			
Evolocumab [16]	Plasma PCSK9	FOURIER	N = 27,564 ASCVD and receiving statin therapy (LDL-C ≥1.81 mmol/L [70 mg/dL])	59%	RRR: 20.3% at 2.2 years
		•Evolocumab (either 140 mg Q2W or 420 mg QM) ± statin and/or ezetimibe			ARR: 1.5 % at 2.2 years
		•Placebo (maximum tolerated statin)			
Alirocumab [17]	Plasma PCSK9	ODYSSEY	N = 18,924 patients with ACS 1–12 months earlier receiving high-intensity statin therapy (LDL-C ≥1.81 mmol/L [70 mg/dL])	54.7%	RRR: 14.4% at 4 ${\mathrm{years}}^{1}$
		•Alirocumab 75 mg			ARR:1.6% at 4 ${\mathrm{years}}^{1}$
		•Placebo (maximum tolerated statin)			
Inclisiran [18, 19]	PCSK9 mRNA	ORION-4	N = ∼15,000 patients with history or evidence of ASCVD receiving maximally tolerated statin therapy	${\mathrm{44.2\%}}^{2}$	Not yet reported
		•Inclisiran 300 mg			
		•Placebo			

^1^The endpoint reported for ODYSSEY is composite of death from coronary heart disease, nonfatal myocardial infarction, fatal or nonfatal ischemic stroke, or unstable angina requiring hospitalization; ^2^Since the results for ORION-4 are not yet available, the 4-year mean LDL-C reduction for inclisiran is reported from ORION-3. ApoB, apolipoprotein B; ARR, absolute risk reduction; ACS, acute coronary syndrome; ASCVD, atherosclerotic cardiovascular disease; ATP, adenosine triphosphate; CV, cardiovascular; FOURIER, **F**urther Cardiovascular **Ou**tcomes **R**esearch With PCSK9 **I**nhibition in Subjects With **E**levated **R**isk; IMPROVE-IT, **IMP**roved **R**eduction of **O**utcomes, **V**ytorin **E**fficacy **I**nternational **T**rial; LDL-C, low-density lipoprotein cholesterol; LLT, lipid-lowering therapy; HMG-CoA, β-hydroxy β-methylglutaryl coenzyme A; MI, myocardial infarction; NPC1L1, Niemann-Pick C1-Like 1; PCSK9, proprotein convertase subtilisin/kexin type 9; mRNA, RNA, messenger ribonucleic acid; RRR, relative risk reduction; TNT, **T**reating to **N**ew **T**argets; Q2W, every 2 weeks; QM, once monthly.

The two available PCSK9i monoclonal antibodies (mAbs), alirocumab and evolocumab, have been approved globally for 8 years at the time of this review. Both have demonstrated remarkable LDL-C reductions of ~60% in adult patients with hyperlipidemia on background statin therapy (± other LLT) in the clinical trial setting [7, 20]. Further, RWE in patients with established ASCVD, and specifically post-acute MI, suggests LLT intensification with a PCSK9i confers significant reduction in LDL-C and a greater proportion of patients achieving below guideline-recommended LDL-C thresholds compared with statins or ezetimibe alone or in combination [21, 22]. However, in a U.S. registry of patients with ASCVD and LDL-C above the goal of <1.8 mmol/L (<70 mg/dL), only 17.1% had LLT intensification 2 years later, with only 3.6% initiated on a PCSK9i [22]. In patients with acute MI, the time to LLT intensification with a PCSK9i tended to be longer than for other therapies, and such patients had a higher ASCVD event rate between their MI and PCSK9i initiation [21], again emphasizing the importance of early and effective intervention for optimal LDL-C management. Additionally, although specific cost-effectiveness analysis will vary by country and healthcare system, a recent Canadian analysis supports the use of PCSK9i in the secondary prevention of ASCVD [23]. The addition of evolocumab to optimized statin therapy ± ezetimibe is associated with an incremental cost per quality-adjusted life year (QALY) gained of $66,453 coronary artery disease (CAD). Moreover, with an incremental cost-effectiveness ratio of $100,000 CAD, the use of evolocumab as an add-on therapy has a 99.9% probability of being cost-effective, at a willingness-to-pay threshold of $100,00 CAD per additional QALY gained. Furthermore, for every 100 patients treated for lifetime, the addition of evolocumab to optimized LLT was estimated to prevent ~52 CV events, of which 7 would be fatal. Hence, the present review aims to summarize the available clinical trial evidence and RWE for evolocumab to inform dyslipidemia treatment decision-making in patient’s requiring intensification of LLT. Overall, the evidence demonstrates the potential of evolocumab to address a healthcare gap in LDL-C lowering and secondary prevention of ASCVD in a variety of high- and very high-risk patients who require additional lipid lowering.

## 2. Evolocumab Mechanism of Action and Clinical Development 

### 2.1 Role of PCSK9 in LDL-C Metabolism

PCSK9 is a serine protease that is predominantly synthesized and secreted by hepatocytes [24]. The only known human function of PCSK9 is to regulate the cell membrane low-density lipoprotein (LDL) receptor (LDLR) in the liver [24]. Following free PCSK9 binding, the LDLR is degraded instead of being recycled, leading to higher circulating LDL-C levels [24]. The potential role of PCSK9 in LDL-C metabolism was first recognized in 2003 via genetic mapping in patients with autosomal dominant hypercholesterolemia [25]. Subsequent case reports of healthy patients homozygous for loss-of-function variants and LDL-C levels of ~0.4 mmol/L (15 mg/dL) demonstrated the crucial role of PCSK9 in LDL metabolism [26]. Indeed, in an analysis of the Atherosclerosis Risk in Communities (ARIC) study in 2006, patients carrying a nonsense, or loss-of-function, variant (specifically either *PCSK9* p.Tyr142Ter or p.Cys679Ter) had 28% lower LDL-C as well as significantly lower total cholesterol and triglycerides compared with non-carriers [27]. During the 15-year follow-up period, only ~1% of carriers experienced a coronary event compared with ~10% of non-carriers [27].

### 2.2 Overview of the Evolocumab Clinical Trial and RWE Program

Several clinical trials studying the safety and efficacy of evolocumab were launched in 2010 (Table 2, Ref. [11, 28, 29, 30, 31, 32]; Table 3, Ref. [11, 33]; Table 4, Ref. [11, 34, 35]; Table 5, Ref. [11, 16, 36]; Table 6, Ref. [11, 37, 38, 39, 40, 41, 42, 43]; Table 7, Ref. [11, 44, 45, 46]) [47], taking evolocumab from bench to bedside in ~7 years. Over the years, the program of evolocumab data generation, known as the Program to Reduce LDL-C and Cardiovascular Outcomes Following Inhibition of PCSK9 In Different Populations (PROFICIO), has grown to include 50 clinical trials and RWE studies to date, enrolling >51,000 patients. [48, 49] Most clinical trials conducted within the PROFICIO program investigated evolocumab added to maximally tolerated statin therapy, compared with a matching injectable subcutaneous placebo and maximally tolerated statin therapy, with background LLT being balanced across evolocumab and control arms. The PROFICIO program has also included a focus on special patient populations, including those with peripheral arterial disease (PAD), diabetes mellitus, recent/prior MI, heterozygous FH (HeFH), homozygous FH (HoFH), pediatric HeFH, and human immunodeficiency virus (HIV), from study sites across the world (Table 6). The results across all trials included support consistent and statistically significant LDL-C reduction with evolocumab across different trial settings and in various patient population. Ultimately, evolocumab was approved in North America and Europe in 2015 as an add-on to statin therapy, alone or in combination with other LLTs, and in those with statin intolerance, for LDL-C reduction in adult patients with primary hyperlipidemia (Table 2) [24]. More recent approvals for evolocumab are for the LDL-C reduction in pediatric patients aged ≥10 years with HeFH or HoFH and for the prevention of CV events (MI, stroke, coronary revascularization) in patients with ASCVD [24].

**Table 2.** **LDL cholesterol reductions with evolocumab in patients with dyslipidemia**.

Study name, publication year	Study rationale	N (*n* on evolocumab)	Trial population	Baseline LDL-C	Background ${\mathrm{LLT}}^{1}$ (n)	Endpoint (Weeks)	Statistically significant (*p* < 0.05) mean LDL-C reduction post-evolocumab vs. ${\mathrm{control}}^{2}$ (%)
MENDEL-2, 2014 [28]	Evaluate 2 evolocumab dosing regimens as monotherapies	614 (306)	Adult patients with hypercholesterolemia (LDL-C ≥2.59 mmol/L [100 mg/dL])	140 mg Q2W: 3.67 mmol/L (142 mg/dL)	None	12	140 mg Q2W: 57.0% 420 mg QM: 56.1%
				420 mg QM: 3.72 mmol/L (144 mg/dL)			
DESCARTES, 2014 [29]	Evaluate longer-term use of evolocumab	901 (599)	Adult patients with hypercholesterolemia (LDL-C ≥1.94 mmol/L [75 mg/dL])	2.69 mmol/L (104.2 mg/dL)	•None: 74	52	${\mathrm{50.1\%}}^{3}$
					•Atorvastatin 10 mg: 254		
					•Atorvastatin 80 mg: 145		
					•Atorvastatin 80 mg + ezetimibe: 126		
LAPLACE-2, 2014 [30]	Evaluate 2 evolocumab dosing regimens in combination with different statin intensities	2067 (1117)	Adult patients with hypercholesterolemia and mixed dyslipidemia (LDL-C ≥2.07 mmol/L [80 mg/dL])	2.97 mmol/L (114.9 mg/dL)	High-intensity statin: 442	12	High-intensity statin patients:
							Atorvastatin 80 mg: 61.8%
							Rosuvastatin 40 mg: 58.9%
					Moderate-intensity statin: 675		Moderate-intensity statin patients:
							Rosuvastatin 5 mg: 60.1%
							Atorvastatin 10 mg: 61.6%
							Simvastatin 40 mg: 65.9%
YUKAWA-2, 2016 [31]	Evaluate 2 evolocumab dosing regimens in combination with atorvastatin in Japanese patients	404 (202)	Adult Japanese patients with hypercholesterolemia/mixed dyslipidemia and high cardiovascular risk (LDL-C ≥2.59 mmol/L [100 mg/dL])	2.82 mmol/L (109 mg/dL)	Atorvastatin: 202 (all patients)	12	140 mg Q2W: 75.9%
							420 mg QM: 66.9%
Phase II pooled analysis (time-averaged), 2022 [32]	Conduct a time-averaged analysis of cumulative LDL-C lowering with evolocumab	372 (189)	Adult patients with hypercholesterolemia	140 mg Q2W: 3.43 mmol/L (132.7 mg/dL)	Statin and/or ezetimibe	9–12	140 mg Q2W: ${\mathrm{67.6\%}}^{3}$
					•Non-intensive statin: 93		
					•Intensive statin: 34		
				420 mg QM: 3.65 mmol/L (141.4 mg/dL)	•Ezetimibe: 14		420 mg QM: ${\mathrm{65.0\%}}^{3}$

^1^Statin intensity was defined based on the 2013 American College of Cardiology/American Heart Association Guideline on the Treatment of Blood Cholesterol to Reduce Atherosclerotic Cardiovascular Risk in Adults [11]; Low intensity statins include atorvastatin (5 mg), lovastatin (20 mg), pravastatin (8.57 mg, 10 mg and 20 mg), rosuvastatin (0.36 mg, 0.71 mg, 1.07 mg, 1.25 mg, 1.43 mg and 2.50 mg) and simvastatin (10 mg); Moderate intensity statins include atorvastatin (10 mg and 20 mg), pravastatin (40 mg), rosuvastatin (5 mg, 10 mg and 15 mg) and simvastatin (20 mg and 40 mg); High intensity statins include atorvastatin (40 mg and 80 mg) and rosuvastatin (20 mg and 40 mg); ^2^Matching injectable subcutaneous placebo and maximally tolerated statin therapy; ^3^*p*-value not reported. DESCARTES, **D**urable **E**ffect of PC**S**K9 Antibody **C**omp**AR**ed Wi**T**h Plac**E**bo **S**tudy; LAPLACE-2, **L**DL-C **A**ssessment With **P**CSK9 Monoclona**L A**ntibody Inhibition **C**ombined With StatinTh**E**rapy-2; LDL-C, low-density lipoprotein-cholesterol; LLT, lipid-lowering therapy; MENDEL-2, Monoclonal Antibody Against PCSK9 to Reduce Elevated LDL-C in Subjects Currently Not Receiving Drug Therapy for Easing Lipid Levels-2; Q2W, every 2 weeks; QM, every month; YUKAWA-2, Stud**Y** of LDL-Cholesterol Reduction **U**sing a Monoclonal PCS**K**9 **A**ntibody in Japanese Patients **W**ith **A**dvanced Cardiovascular Risk; PCSK9, proprotein convertase subtilisin-kexin type 9; LDL, low-density lipoprotein.

**Table 3.** **LDL cholesterol reductions with self administration of evolocumab**.

Study name, publication year	Study rationale	N (*n* on evolocumab)	Trial population	Baseline LDL-C	Background ${\mathrm{LLT}}^{1}$ (n)	Endpoint (Weeks)	Statistically significant (*p* < 0.05) mean LDL-C reduction post-evolocumab vs. ${\mathrm{control}}^{2}$ (%)
THOMAS-1, 2016 [33]	Evaluate users’ ability to self-administer evolocumab in a home-use setting	149 (149)	Adult patients with hypercholesterolemia or mixed dyslipidemia (LDL-C ≥2.20 mmol/L [85 mg/dL])	3.02–3.05 mmol/L (116.9–118.1 mg/dL)	Statin ± ezetimibe	6	${\mathrm{63.4\%}}^{3}$
					•Statin: 149 (all patients)		
					•Ezetimibe: 9		
THOMAS-2, 2016 [33]	Evaluate users’ ability to self-administer evolocumab in a home-use setting	164 (164)	Adult patients with hypercholesterolemia or mixed dyslipidemia (LDL-C ≥2.20 mmol/L [85 mg/dL])	2.98–3.03 mmol/L (115.3–117.3 mg/dL)	Statin ± ezetimibe	Mean of weeks 10 and 12	${\mathrm{67.9\%}}^{3}$
					•Statin: 164 (all patients)		
					•Ezetimibe: 14		

^1^Statin intensity was defined based on the 2013 American College of Cardiology/American Heart Association Guideline on the Treatment of Blood Cholesterol to Reduce Atherosclerotic Cardiovascular Risk in Adults [11]; Low intensity statins include atorvastatin (5 mg), lovastatin (20 mg), pravastatin (8.57 mg, 10 mg and 20 mg), rosuvastatin (0.36 mg, 0.71 mg, 1.07 mg, 1.25 mg, 1.43 mg and 2.50 mg) and simvastatin (10 mg); Moderate intensity statins include atorvastatin (10 mg and 20 mg), pravastatin (40 mg), rosuvastatin (5 mg, 10 mg and 15 mg) and simvastatin (20 mg and 40 mg); High intensity statins include atorvastatin (40 mg and 80 mg) and rosuvastatin (20 mg and 40 mg); ^2^Matching injectable subcutaneous placebo and maximally tolerated statin therapy; ^3^*p*-value not reported. LDL-C, low-density lipoprotein-cholesterol; LLT, lipid-lowering therapy; LDL, low-density lipoprotein.

**Table 4.** **LDL cholesterol reductions and impact on plaque imaging/morphology with evolocumab**.

Study name, publication year	Study rationale	N (*n* on evolocumab)	Trial population	Baseline LDL-C	Background ${\mathrm{LLT}}^{1}$ (n)	Endpoint (Weeks)	Statistically significant (*p* < 0.05) mean LDL-C reduction post-evolocumab vs. ${\mathrm{control}}^{2}$ (%)
GLAGOV, 2016 [34]	Evaluate whether LDL-C lowering with evolocumab results in greater change from baseline in PAV	968 (484)	Adult patients with coronary angiography (LDL-C ≥1.55 mmol/L [60 mg/dL])	2.39 mmol/L (92.5 mg/dL)	•High-intensity statin: 280	78	${\mathrm{60.8\%}}^{3}$
					•Moderate-intensity statin: 196		
					•Low-intensity statin: 2		
					•Ezetimibe: 9		
HUYGENS, 2022 [35]	Evaluate whether evolocumab inhibition in addition to high-intensity statin therapy favorably modifies coronary plaque phenotype	164 (80)	Adult patients with non-ST segment elevation MI (LDL-C ≥1.55 mmol/L [60 mg/dL])	3.62 mmol/L (140.0 mg/dL)	Statin and/or ezetimibe	50	${\mathrm{81.4\%}}^{3}$
					•High-intensity statin: 63		
					•Moderate-intensity statin: 11		
					•Low-intensity statin: 1		
					•Ezetimibe: 1		

^1^Statin intensity was defined based on the 2013 American College of Cardiology/American Heart Association Guideline on the Treatment of Blood Cholesterol to Reduce Atherosclerotic Cardiovascular Risk in Adults [11]; Low intensity statins include atorvastatin (5 mg), lovastatin (20 mg), pravastatin (8.57 mg, 10 mg and 20 mg), rosuvastatin (0.36 mg, 0.71 mg, 1.07 mg, 1.25 mg, 1.43 mg and 2.50 mg) and simvastatin (10 mg); Moderate intensity statins include atorvastatin (10 mg and 20 mg), pravastatin (40 mg), rosuvastatin (5 mg, 10 mg and 15 mg) and simvastatin (20 mg and 40 mg); High intensity statins include atorvastatin (40 mg and 80 mg) and rosuvastatin (20 mg and 40 mg); ^2^Matching injectable subcutaneous placebo and maximally tolerated statin therapy; ^3^Percentage LDL-C reduction calculated based on absolute change in LDL-C at the end of the study compared to baseline. GLAGOV, **GL**obal **A**ssessment of Plaque Re**G**ression with a PCSK9 Antib**O**dy as Measured by Intra**V**ascular Ultrasound; HUYGENS, **H**igh-Resol**U**tion Assessment of Coronar**Y** Plaques in a **G**lobal **E**volocumab Ra**N**domized **S**tudy; LDL-C, low-density lipoprotein-cholesterol; LLT, lipid-lowering therapy; MI, myocardial infarction; PAV, percent atheroma volume; LDL, low-density lipoprotein; PCSK9, proprotein convertase subtilisin-kexin type 9.

**Table 5.** **LDL cholesterol reductions with evolocumab in cardiovascular outcomes trials**.

Study name, publication year	Study rationale	N (*n* on evolocumab)	Trial population	Baseline LDL-C	Background ${\mathrm{LLT}}^{1}$ (n)	Endpoint (Weeks)	Statistically significant (*p* < 0.05) mean LDL-C reduction post-evolocumab vs. ${\mathrm{control}}^{2}$ (%)
FOURIER, 2017 [16]	Evaluate the effect of evolocumab on the risk of CV death, MI, stroke, hospitalization for unstable angina, or coronary revascularization	27,564 (13,784)	Adult patients with ASCVD and LDL-C ≥1.8 mmol/L (70 mg/dL)	2.4 mmol/L (92 mg/dL)	Statin and/or ezetimibe	48	59%
					•High-intensity statin: 9585		
					•Moderate-intensity statin: 4161		
					•Low- or unknown intensity statin: 38		
					•Ezetimibe: 726		
FOURIER-OLE, 2022 [36]	Evaluate long-term safety, tolerability, lipids levels, and risk of major adverse cardiovascular events with continued evolocumab exposure	6635 (3355)	Adult patients with ASCVD and LDL-C ≥1.8 mmol/L (70 mg/dL)	2.35 mmol/L (91 mg/dL) in parent FOURIER	Statin and/or ezetimibe	12	${\mathrm{58.4\%}}^{3}$
					•High-intensity statin: 2584		
					•Moderate-intensity statin: 758		
					•Low- or unknown statin intensity: 13		
					•Ezetimibe: 200		

^1^Statin intensity was defined based on the 2013 American College of Cardiology/American Heart Association Guideline on the Treatment of Blood Cholesterol to Reduce Atherosclerotic Cardiovascular Risk in Adults [11]; Low intensity statins include atorvastatin (5 mg), lovastatin (20 mg), pravastatin (8.57 mg, 10 mg and 20 mg), rosuvastatin (0.36 mg, 0.71 mg, 1.07 mg, 1.25 mg, 1.43 mg and 2.50 mg) and simvastatin (10 mg); Moderate intensity statins include atorvastatin (10 mg and 20 mg), pravastatin (40 mg), rosuvastatin (5 mg, 10 mg and 15 mg) and simvastatin (20 mg and 40 mg); High intensity statins include atorvastatin (40 mg and 80 mg) and rosuvastatin (20 mg and 40 mg); ^2^Matching injectable subcutaneous placebo and maximally tolerated statin therapy; ^3^*p*-value not reported. ASCVD, atherosclerotic cardiovascular disease; CV, cardiovascular; FOURIER, **F**urther Cardiovascular **Ou**tcomes **R**esearch With PCSK9 **I**nhibition in Subjects With **E**levated **R**isk; LDL-C, low-density lipoprotein-cholesterol; LLT, lipid-lowering therapy; MI, myocardial infarction; OLE, open label extension; LDL, low-density lipoprotein; PCSK9, proprotein convertase subtilisin-kexin type 9.

**Table 6.** **LDL cholesterol reductions with evolocumab in special populations**.

Study name, publication year	Study rationale	N (*n* on evolocumab)	Trial population	Baseline LDL-C	Background ${\mathrm{LLT}}^{1}$ (n)	Endpoint (Weeks)	Statistically significant (*p* < 0.05) mean LDL-C reduction post-evolocumab vs. ${\mathrm{control}}^{2}$ (%)
RUTHERFORD-2, 2015 [37]	Evaluate 2 dosing regimens of evolocumab in subjects with HeFH	331 (221)	Adult patients with HeFH (LDL-C ≥2.59 mmol/L [100 mg/dL])	140 mg Q2W:	Statin and/or ezetimibe: 221 (all patients)	12	140 mg Q2W: 61.3%
				4.2 mmol/L (162.4 mg/dL)			
				420 mg QM:			420 mg QM: 55.7%
				4.0 mmol/L (154.68 mg/dL)			
GAUSS-3, 2016 [38]	Compare effectiveness and tolerability of evolocumab and ezetimibe in patients with statin-induced muscle symptoms	491 (145)	Adult patients with a history of statin intolerance and not at LDL-C goal (LDL-C ≥2.59 mmol/L [100 mg/dL])	5.66 mmol/L (218.8 mg/dL)	None	24	52.8%
BANTING, 2019 [39]	Evaluate the effect of evolocumab in adults with type 2 diabetes mellitus and high cholesterol	421 (281)	Adult patients with type 2 diabetes and hypercholesterolemia/mixed dyslipidemia (variable LDL-C criteria)	2.81 mmol/L (108.7 mg/dL)	•High-intensity statin: 146	12	54.3%
					•Moderate-intensity statin: 133		
EVOPACS, 2019 [40]	Evaluate evolocumab administered in-hospital in patients presenting with ACS	308 (155)	Adult patients hospitalized for acute coronary syndromes with elevated LDL-C beyond guideline-recommended target	3.61 mmol/L (139.6 mg/dL)	Statin and/or ezetimibe	8	77.1%
					•High-intensity statin: 18		
					•Low- or moderate-intensity statin: 13		
					•No statin: 124		
					•Ezetimibe: 6		
HAUSER, 2020 [41]	Evaluate safety and efficacy of evolocumab in pediatric subjects aged 10–17 years diagnosed with HeFH	157 (104)	Pediatric patients (10–17 years) with HeFH (LDL-C ≥3.4 mmol/L [130 mg/dL])	4.78 mmol/L (185.0 mg/dL)	Statin and/or ezetimibe	24	44.5%
					•High-intensity statin: 19		
					•Moderate-intensity statin: 63		
					•Low- or unknown intensity statin: 22		
					•Ezetimibe: 13		
EVACS, 2020 [42]	Evaluate impact of evolocumab on early postinfarct atherogenic lipoprotein trajectories in patients with ACS	57 (30)	Adult patients with non-ST segment elevation MI and troponin I ≥5 ng/mL	2.37 mmol/L (91.5 mg/dL)	•All patients received high-intensity statin unless contraindicated	4 (day 30)	${\mathrm{31\%}}^{3}$
BEIJERINCK, 2020 [43]	Evaluate evolocumab efficacy and tolerability in HIV-positive patients	464 (310)	Adult patients with HIV with hypercholesterolemia/mixed dyslipidemia (LDL-C ≥2.59 mmol/L [100 mg/dL])	3.45 mmol/L (133.3 mg/dL)	None, or statin and/or ezetimibe	24	56.9%
					•High-intensity statin: 95		
					•Moderate-intensity statin: 137		
					•No statin: 61		
					•Ezetimibe: 53		

^1^Statin intensity was defined based on the 2013 American College of Cardiology/American Heart Association Guideline on the Treatment of Blood Cholesterol to Reduce Atherosclerotic Cardiovascular Risk in Adults [11]; Low intensity statins include atorvastatin (5 mg), lovastatin (20 mg), pravastatin (8.57 mg, 10 mg and 20 mg), rosuvastatin (0.36 mg, 0.71 mg, 1.07 mg, 1.25 mg, 1.43 mg and 2.50 mg) and simvastatin (10 mg); Moderate intensity statins include atorvastatin (10 mg and 20 mg), pravastatin (40 mg), rosuvastatin (5 mg, 10 mg and 15 mg) and simvastatin (20 mg and 40 mg); High intensity statins include atorvastatin (40 mg and 80 mg) and rosuvastatin (20 mg and 40 mg); ^2^Matching injectable subcutaneous placebo and maximally tolerated statin therapy; ^3^Percentage LDL-C reduction calculated based on absolute change in LDL-C at the end of the study compared to baseline. ACS, acute coronary syndrome; EVACS, **EV**olocumab in **A**cute **C**oronary **S**yndrome; EVOPACS, **EVO**locumab for Early Reduction of LDL-Cholesterol Levels in **P**atients With **A**cute **C**oronary **S**yndromes; GAUSS-3, **G**oal **A**chievement After **U**tilizing an Anti-PCSK9 Antibody in **S**tatin Intolerant; HAUSER, Trial Assessing Efficacy, Safety and Tolerability of PCSK9 In**H**ibition in Pedi**A**tric S**U**bject**S **With Gen**E**tic LDL Disorde**R**s; HeFH, heterozygous familial hypercholesterolemia; HIV, human immunodeficiency virus; LDL-C, low-density lipoprotein-cholesterol; LLT, lipid-lowering therapy; MI, myocardial infarction; Q2W, every 2 weeks; QM, every month; RUTHERFORD-2, **R**ed**U**ction of LDL-C With PCSK9 Inhibi**T**ion in **HE**te**R**ozygous **F**amilial Hyperch**O**leste**R**olemia **D**isorder Study-2; LDL, low-density lipoprotein; PCSK9, proprotein convertase subtilisin-kexin type 9; BANTING, Evolocuma**B** Effic**A**cy a**N**d Safe**T**y **IN** Type 2 Diabetes Mellitus on Back**G**round Statin Therapy; BEIJERINCK, Evolocuma**B E**ffect on LDL-C Lowering in Sub**JE**cts with Human Immunodeficiency Vir**R**us and **IN**creased **C**ardiovascular Ris**K**.

**Table 7.** **LDL cholesterol reductions with evolocumab in select real-world studies**.

Study name, publication year	Study rationale	N (*n* on evolocumab)	Trial population	Baseline LDL-C	Background ${\mathrm{LLT}}^{1}$ (n)	Endpoint (Weeks)	Statistically significant (*p* < 0.05) mean LDL-C reduction post-evolocumab vs. ${\mathrm{control}}^{2}$ (%)
HEYMANS, 2022 [44] & 2023 [45]	Review evolocumab effectiveness and safety in European patients in a real-world setting	1951 (all patients)	Adult hyperlipidemic patients receiving evolocumab (LDL-C criteria varied based on region)	3.98 mmol/L (153.9 mg/dL)	•Neither statin, nor ezetimibe: 799	12 (3 months)	${\mathrm{58\%}}^{3,4}$
					•Any statin: 840		
					•Statin without ezetimibe: 234		
					•Statin with ezetimibe: 605		
					•Ezetimibe without statin: 312		
ZERBINI, 2023 [46]	Review evolocumab effectiveness and safety in patients across Canada, Mexico, Colombia, Saudi Arabia and Kuwait in a real-world setting	578 (all patients)	Adult hyperlipidemic patients receiving evolocumab (LDL-C ≥1.8 mmol/L [70 mg/dL])	3.4 mmol/L (131.5 mg/dL)	•Statin: 437	Up to 52 (12 months)	${\mathrm{70.2\%}}^{4,5}$
					•Ezetimibe without statin: 39		
					•Ezetimibe + statin: 168		
					•Bile acid sequestrant: 16		
					•Other LLT (EPACOR, fibrates, niacin): 25		

^1^Statin intensity was defined based on the 2013 American College of Cardiology/American Heart Association Guideline on the Treatment of Blood Cholesterol to Reduce Atherosclerotic Cardiovascular Risk in Adults [11]; Low intensity statins include atorvastatin (5 mg), lovastatin (20 mg), pravastatin (8.57 mg, 10 mg and 20 mg), rosuvastatin (0.36 mg, 0.71 mg, 1.07 mg, 1.25 mg, 1.43 mg and 2.50 mg) and simvastatin (10 mg); Moderate intensity statins include atorvastatin (10 mg and 20 mg), pravastatin (40 mg), rosuvastatin (5 mg, 10 mg and 15 mg) and simvastatin (20 mg and 40 mg); High intensity statins include atorvastatin (40 mg and 80 mg) and rosuvastatin (20 mg and 40 mg); ^2^Matching injectable subcutaneous placebo and maximally tolerated statin therapy; ^3^Median LDL-C reduction reported; ^4^Percentage LDL-C reduction calculated based on absolute change in LDL-C at the end of the study compared to baseline; ^5^*p*-value not reported. HEYMANS, C**H**aract**E**ristics of H**Y**perlipidae**M**ic P**A**tie**N**ts at Initiation of Evolocumab and Treatment Pattern**S**; LDL-C, low-density lipoprotein-cholesterol; LLT, lipid-lowering therapy; ZERBINI, Multi**Z**onal Obs**ER**vational Study Conducted **B**y Clin**I**cal Practitioners on Evolocumab Use i**N **Subjects With Hyperlip**I**demia; LDL, low-density lipoprotein.

### 2.3 Evolocumab Dosing and Administration

Evolocumab is administered subcutaneously, with a recommended dose of either 140 mg every 2 weeks (Q2W) or 420 mg once monthly (QM) [20]. It can be administered using a prefilled syringe or prefilled autoinjector, and is intended for patient self-administration or administration by a caregiver [20]. In the phase III **T**rial for **HOM**e-use of prefilled **A**uto-injector pen and 3.5 mL Personal Injector in AMG 145 administration**S** (THOMAS)-I and THOMAS-II studies (Table 3), patients were confirmed to be successful at self-administering evolocumab in both the clinic and at-home settings, regardless of the dosing schedule or injection device [33].

### 2.4 Evolocumab Pharmacokinetics/Pharmacodynamics (PK/PD)

Following a single subcutaneous dose of evolocumab (140 mg or 420 mg) administered to healthy adults, peak circulating drug concentrations are reached in 3–4 days, with an estimated absolute bioavailability of 72% and half-life of 11–17 days [20, 50]. Evolocumab exerts even more rapid pharmacodynamic effects, with 100% PCSK9 suppression within 4 hours of administration and reductions in LDL-C observed as early as day 1 in clinical trials [20, 42, 50]. Following a single 420 mg intravenous (IV) dose of evolocumab, the mean systemic (± standard deviation) clearance is estimated to be 12 ± 2 mL/hr, with co-administration of statins increasing the clearance of evolocumab by ~20% [20]. Peak LDL-C reduction is generally observed at 1–2 weeks after evolocumab administration, with mean reductions ranging from 50–81% in the clinical trial setting (Tables 2,3,4,5,6,7) [50]. Patient characteristics, including mild/moderate hepatic impairment, kidney impairment or failure, body weight, race, sex, or age, do not contribute to clinically meaningful differences in pharmacodynamic effects of evolocumab on LDL-C reduction [50]. Further, PCSK9i mAbs are very specific, including evolocumab which binds to PCSK9 with a high affinity of 16 pM, and they are not known to bind to other members of the PCSK enzyme superfamily [51]. Across 17,992 adults patients treated with evolocumab in clinical studies, only 0.3% tested positive for the development of anti-evolocumab binding antibodies, with none of these patients developing neutralizing antibodies [20]. Finally, no adverse drug-drug interactions have been reported for evolocumab to date [20].

## 3. Effect of Evolocumab on Coronary Plaque

### 3.1 Impact of Evolocumab on Plaque Burden 

**Fig. 1.**
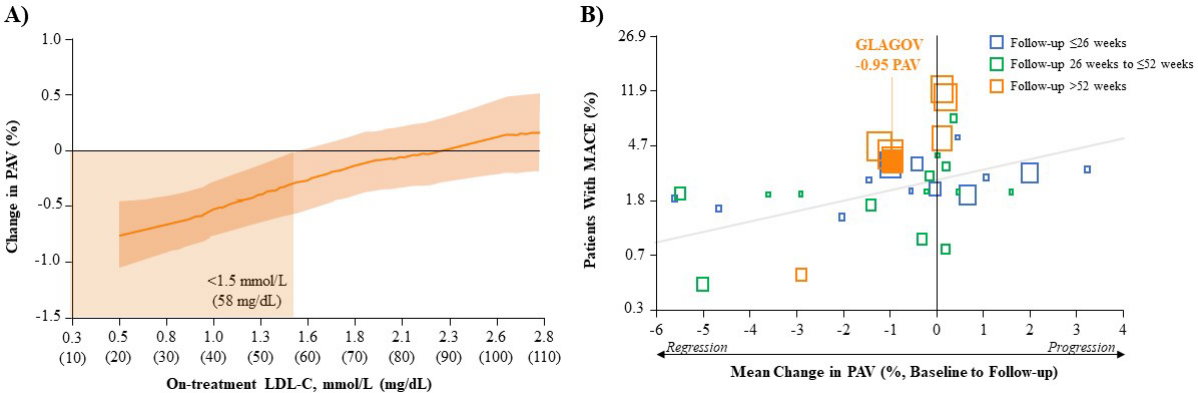
**Correlation of LDL-C reduction, change in PAV and MACE outcomes**. (A) Post-hoc Analysis of the Relationship Between Achieved LDL-C and Change in PAV After Evolocumab (+Statin) Treatment in GLAGOV. Local regression (LOESS) curve illustrating post-hoc analysis of the association (with 95% confidence intervals) between the achieved LDL-C levels and change in PAV in all patients undergoing serial IVUS evaluation. Curve truncated at 0.5 mmol/L and 2.8 mmol/L (20 and 110 mg/dL) owing to the small numbers of values outside that range. (Figure permission obtained from [34]). (B) Association Between Mean Change in PAV and MACE in Various LLT Trials. Each square represents a single study arm. The size of the square is proportional to the sample size of that study arm. The MACE proportion was converted to log odds and a constant 0.5 was added to zero counts to allow the conversion of log odds. The regression line is based on the adjusted mixed-effects logistic regression model. (Figure permission obtained from [53]). GLAGOV, **GL**obal **A**ssessment of Plaque Re**G**ression with a PCSK9 Antib**O**dy as Measured by Intra**V**ascular Ultrasound; IVUS, intravascular ultrasound; LDL-C, low-density lipoprotein cholesterol; LLT, lipid-lowering therapy; MACE, major adverse cardiovascular event (myocardial infarction, stroke, transient ischemic attack, unstable angina or all-cause mortality); PAV, percent atheroma volume.

In addition to marked reductions in LDL-C, imaging studies have demonstrated the efficacy of evolocumab at the level of coronary plaque [34, 35, 52]. In the phase III **GL**obal **A**ssessment of Plaque Re**G**ression with a PCSK9 Antib**O**dy as Measured by Intra**V**ascular Ultrasound (GLAGOV) (Table 4) study in patients with angiographic CAD, LDL-C reductions from baseline after 78 weeks of evolocumab (+statin) were linearly associated with reductions in the percent atheroma (atherosclerotic plaque) volume (PAV) (Fig. 1A, Ref. [34]). The clinical significance of this is clear from a comprehensive systematic review and meta-regression analysis of LLT trials representing data from over 6000 patients, including some on evolocumab, which showed that for every 1% reduction in mean PAV, there is a ~20% reduction in the risk of major CV events (Fig. 1B) [53]. In the GLAGOV study, evolocumab (+statin) reduced PAV by an absolute 0.95% from baseline and resulted in a greater proportion of patients with plaque regression compared with placebo (+statin; 64.3% vs. 47.3%, respectively) [34]. Interestingly, further analysis showed that achieving LDL-C <1.5 mmol/L (<58 mg/dL) resulted in plaque regression, deceleration of progression, or lack of progression (Fig. 1A), all of which were associated with plaque stabilization and reduced CV risk [54]. Likewise, in the phase III **H**igh-Resol**U**tion Assessment of Coronar**Y** Plaques in a **G**lobal **E**volocumab Ra**N**domized **S**tudy (HUYGENS) study in patients with a non-ST-segment elevation MI (NSTEMI), 52 weeks of evolocumab (+statin) therapy resulted in even greater reductions in PAV compared with placebo (+statin; –2.3% vs. –0.6%, respectively) [35].

### 3.2 Impact of Evolocumab on Plaque Stability 

Plaque stability can be characterized by the thickness of the thin-cap fibroatheroma, with an inverse relationship between the thickness of the fibrous cap covering the lipid plaque and risk of plaque rupture [55]. Plaques at high risk of rupture have a fibrous cap thickness <65 µm [56]; therefore, the ability to improve plaque stability in a vulnerable high-risk patient may reduce the risk of plaque rupture and CV events. In the HUYGENS study (Table 4) of NSTEMI patients, evolocumab (+statin) increased the minimum fibrous cap thickness at week 50 from baseline by +42.7 ± 10.3 µm vs. +21.5 ± 10.6 µm in the placebo (+statin) group [35], representing an 81.8% vs. 44.3% improvement, respectively. A greater proportion of patients achieved a minimum fibrous cap thickness ≥65 µm in the evolocumab group compared with the placebo group [35]. Likewise, in the phase IV Reduction in **YE**llow Plaque by Aggressive **L**ipid **LOW**ering Therapy (YELLOW)-III study in patients with stable CAD, evolocumab (+statin) increased fibrous cap thickness from 70.9 ± 21.7 µm at baseline to 97.7 ± 31.1 µm at week 26 [52]. Collectively, these results point to relatively rapid improvements in plaque stability to reduce the risk of rupture as a potential mechanism underlying the clinical benefits of evolocumab, regardless of ASCVD severity.

## 4. Evolocumab Efficacy in Cardiovascular Outcomes Trials

### 4.1 FOURIER

The potential for the effects of evolocumab on LDL-C to translate into CV benefits was explored in the phase III FOURIER trial (Table 5), the largest dedicated CV outcomes trial with a LLT to date (N = 27,564) [16]. FOURIER was an event driven trial that investigated the efficacy and safety of evolocumab vs. placebo added to high- or moderate-intensity statin therapy in patients with clinically evident ASCVD with an LDL-C >1.8 mmol/L (70 mg/dL) or a non-HDL-C >2.6 mmol/L (100 mg/dL), with a median follow-up of 2.2 years. At 48 weeks, evolocumab reduced LDL-C by 59% compared with placebo, which was maintained over time. Further, LDL-C was reduced below thresholds of ≤1.8 mmol/L (≤70 mg/dL) in 87% of patients and ≤1.0 mmol/L (≤40 mg/dL) in 67% of patients on evolocumab, compared with 18% and 0.5% of patients on placebo, respectively. A post-hoc analysis of the interindividual variation in LDL-C reductions from baseline during the first year post-evolocumab initiation in FOURIER showed 94.7% of patients achieved ≥50% LDL-C reduction, 97.9% achieved ≥30% reduction and 99.5% achieved any reduction in LDL-C [57]. Evolocumab also significantly reduced concentrations of other atherogenic lipoproteins, including non-high-density lipoprotein C (HDL-C), apolipoprotein B-100 (ApoB), and lipoprotein (a; Lp(a)), compared with placebo (Table 8, Ref. [16]). A pre-planned analysis of the FOURIER results showed patients with higher baseline Lp(a) levels had greater absolute reductions in Lp(a) and CV risk post-evolocumab treatment, with a 23% reduction in the risk of coronary heart disease death, MI or urgent coronary revascularization compared with only 7% in patients with lower baseline Lp(a) [58].

**Table 8.** **Reduction in additional lipid parameters with evolocumab (+statin) therapy at 48 weeks in the FOURIER trial [16]**.

Lipid parameter	Mean change from baseline, %		Evolocumab vs. Placebo, %	*p*-value
	Placebo	Evolocumab		
LDL-C	NR	NR	–59.0	<0.001
Non-HDL-C	0.4	–51.2	–51.6	<0.001
Triglycerides	–0.7	–16.2	–15.5	<0.001
ApoB	2.7	–46.0	–48.7	<0.001
Lp(a)	0.0	–26.9	–26.9	<0.001

ApoB, apolipoprotein B; FOURIER, **F**urther Cardiovascular **Ou**tcomes **R**esearch With PCSK9 **I**nhibition in Subjects With **E**levated **R**isk; HDL-C, high-density lipoprotein cholesterol; LDL-C, low-density lipoprotein cholesterol; Lp(a), lipoprotein(a); NR, not reported; PCSK9, proprotein convertase subtilisin-kexin type 9.

**Fig. 2.**
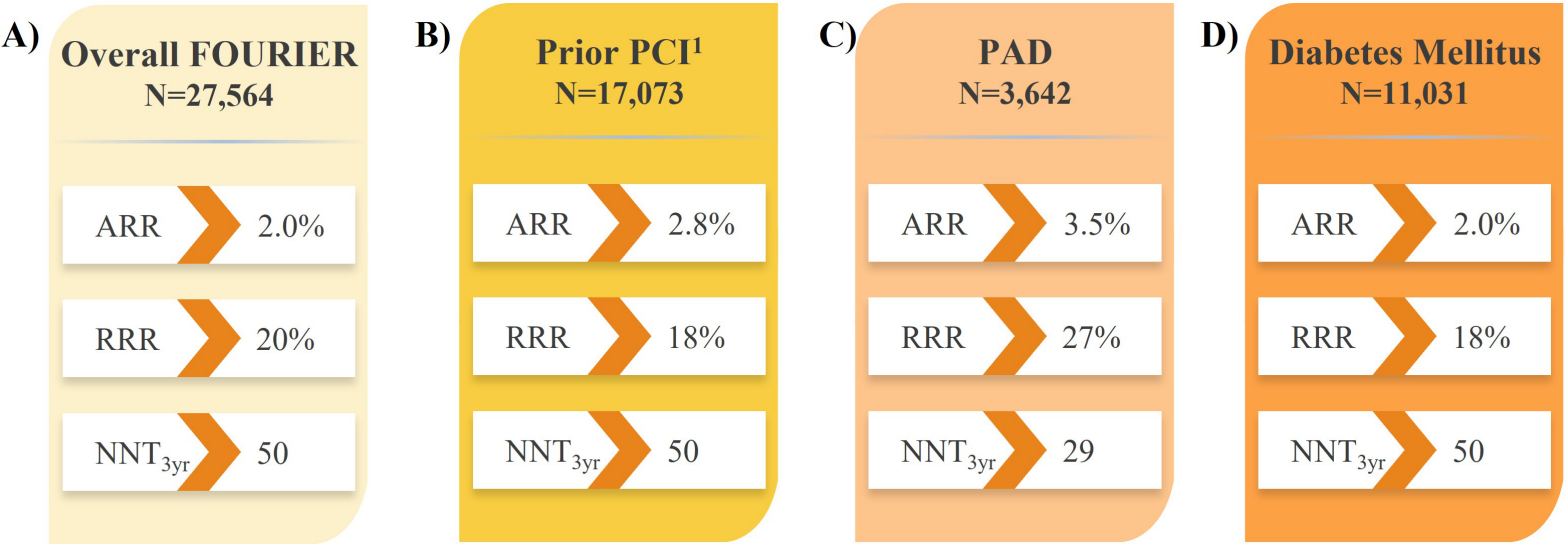
**Impact of evolocumab vs. placebo on CV death, MI, or stroke in high- and very high-risk patients in the FOURIER trial**. (A) Overall FOURIER [16]. (B) Prior PCI [63]. (C) PAD [64]. (D) Diabetes Mellitus [65]. ^1^Composite of coronary death, MI, or coronary revascularization. ARR, absolute risk reduction; CV, cardiovascular; FOURIER, **F**urther Cardiovascular **Ou**tcomes **R**esearch With PCSK9 **I**nhibition in Subjects With **E**levated **R**isk; NNT, number needed to treat; PAD, peripheral arterial disease; PCI, percutaneous coronary intervention; RRR, relative risk reduction; MI, myocardial infarction.

Findings from the FOURIER trial highlighted the benefit of reducing LDL-C to levels lower than those shown in previous LLT trials, with a median achieved LDL-C of 0.78 mmol/L (30.2 mg/dL) [16]. Importantly, this significant LDL-C reduction with evolocumab resulted in reduced CV events which can be visualized in the Kaplan-Meier curved from the FOURIER, which showcase a 15% reduction in the risk of the prespecified primary composite endpoint of CV death, MI, stroke, hospitalization for unstable angina, or coronary revascularization (Fig. 2A, Ref. [16]), and a 20% reduction in the risk of the key secondary composite endpoint of major adverse CV events (MACE; i.e., CV death, MI, or stroke; Fig. 2B, Ref. [16]). Differences in CV risk between the evolocumab and placebo groups were observed early, at approximately 6 months, and increased over time. The Kaplan-Meier curves from the FOURIER analysis demonstrate the magnitude of MACE risk reduction was 16% during the first year that increased to 25% beyond the first year post-evolocumab initiation, collectively highlighting a rapid onset of MACE risk reduction that grew over time, considering the relatively short duration of follow-up (**Supplementary Fig. 4** in [16]). Further, MACE risk reductions were consistent across levels of background statin intensity and ezetimibe use and regardless of baseline LDL-C. Among patients in the top quartile for baseline LDL-C (n = 6829), evolocumab reduced median LDL-C from 3.3 mmol/L (126 mg/dL) to 1.1 mmol/L (43 mg/dL) and the risk of MACE by 17%. Among patients in the lowest quartile for baseline LDL-C (n = 6961), evolocumab reduced median LDL-C from 1.9 mmol/L (73 mg/dL) to 0.57 mmol/L (22 mg/dL) and the risk of MACE by 22%. Further, in a subset of patients who had baseline LDL-C <1.8 mmol/L (<70 mg/dL), a significant 30% reduction in MACE was shown, reinforcing the importance of further LDL-C reduction even in high-risk ASCVD patients who are close to the recommended LDLC threshold [59]. Finally, evolocumab had no impact on any treatment emergent adverse events apart from a small increase in local injection site reactions [16], even at the lowest achieved LDL-C levels [59], as described in detail in section 9 on evolocumab safety.

### 4.2 FOURIER-OLE

An open-label extension of the FOURIER trial (FOURIER-OLE; Table 5) was conducted to determine the long-term efficacy and safety of evolocumab [36]. Patients (N = 6635) who completed FOURIER continued or transitioned to open-label evolocumab (140 mg Q2W or 420 mg QM), with an additional median follow-up of 5.0 years. Maximum exposure to evolocumab in the parent trial plus FOURIER-OLE was 8.4 years, and patients were advised to continue other background LLT whenever appropriate during follow-up. Consistent with the parent FOURIER trial, at 12 weeks after the start of FOURIER-OLE, LDL-C was reduced by 58.4% to a median of 0.75 mmol/L (30 mg/dL), which was consistent between patients irrespective of their original treatment in the parent trial (i.e., evolocumab or placebo). An LDL-C <1.8 mmol/L (<70 mg/dL) was achieved by 87.3% of patients, <1.4 mmol/L (<55 mg/dL) by 80.3% and <1.0 mmol/L (<40 mg/dL) by 63.2%. Further, for patients randomized to evolocumab in the parent FOURIER trial, LDL-C reductions were consistent and stable over a median follow-up of 7.1 years, with no attenuation of effect or fluctuations over time. Accordingly, these patients had a 20% reduced risk of CV death, MI, or stroke and 23% reduced risk of CV death (Table 9, Ref. [16, 36]) compared with patients originally randomized to placebo who were delayed in evolocumab initiation by approximately 2 years until the start of the OLE. The Kaplan-Meier curves for percentage of patients experiencing CV death, MI or stroke (**Supplementary Fig. 4B** in [36]) demonstrate the significant CV benefits associated with both earlier and longer evolocumab intervention [36]. The differences in CV outcomes based on duration of evolocumab exposure may be due to an accrued benefit of prior LDL-C reductions in patients originally randomized to evolocumab, in combination with the lag time required for LDL-C reductions to affect plaque burden in patients originally randomized to placebo and transitioned to evolocumab.

**Table 9.** **CV outcomes in the parent FOURIER + OLE trials**.

Trial	N	CV death, MI, or stroke, %			HR (95% CI)	*p*-value
		Placebo	Placebo → evolocumab	Evolocumab		
Parent FOURIER (Median 2.2 years) [16]	27,564	9.9	-	7.9	0.80 (0.73–0.88)	<0.001
FOURIER OLE (Median 5 years) [36]	6635	-	19.26	16.82	0.80 (0.68–0.93)	0.003
		CV death, %				
Parent FOURIER (Median 2.2 years) [16]	27,564	1.7	-	1.8	1.05 (0.88–1.25)	0.62
FOURIER OLE (Median 5 years) [36]	6635	-	6.87	6.35	0.77 (0.60–0.99)	0.04

CI, confidence interval; CV, cardiovascular; FOURIER, **F**urther Cardiovascular **Ou**tcomes **R**esearch With PCSK9 **I**nhibition in Subjects With **E**levated **R**isk; HR, hazard ratio; MI, myocardial infarction; N, total number of patients included in the trial; OLE, open label extension; PCSK9, proprotein convertase subtilisin-kexin type 9.

In another recent analysis of the FOURIER-OLE with a maximum follow-up of 8.6 years, there was a monotonic relationship between achieved LDL-C levels and CV risk, with every 1 mmol/L (40 mg/dL) reduction in LDL-C conferring ~20% reduction in the risk of major CV events [60]. While this finding is consistent with reports of agents from other LLT trials [7], the FOURIER-OLE uniquely confirmed these CV benefits in patients with LDL-C reductions to lower levels than previously studied, down to <0.5 mmol/L (<20 mg/dL), with no apparent level below which there is no further CV risk reduction [60]. Indeed, patients who achieved LDL-C levels well below guideline-recommended thresholds (<1.0 and <0.5 mmol/L; <40 and <20 mg/dL) tended to have a reduced risk of MACE compared with patients who achieved LDL-C levels within these thresholds (1.4–1.8 mmol/L; 55–70 mg/dL). These results reinforce the importance of targeting very low LDL-C levels in high-risk patients with ASCVD and suggest the most intensive European guideline-recommended LDL-C target threshold of <1.0 mmol/L (<40 mg/dL) could benefit all patients with ASCVD, not just those with recurrent CV events. Further, no new adverse events emerged, even in patients with the lowest achieved LDL-C levels [36, 60], as described in section 9, evolocumab safety.

Altogether, the FOURIER and FOURIER-OLE provide the largest and longest follow-up data available to date for a PCSK9i in patients with ASCVD. The results demonstrate rapid, clinically significant, and sustained efficacy with long-term evolocumab (+statin) treatment, with compounding CV benefits at lower achieved LDL-C levels and over time. Hence, the results emphasize the importance of early and significant LDL-C reduction to achieve the greatest clinical outcomes.

## 5. Evolocumab Efficacy in Vulnerable Patient Populations with Increased Risk of CV Events (ACS, Prior MI, Prior PCI, PAD, Diabetes Mellitus, Metabolic Syndrome)

**Fig. 3.**
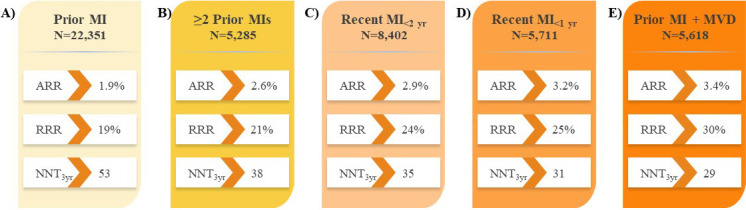
**Impact of evolocumab on CV death, MI, or stroke in patients with prior MI in the FOURIER trial**. (A) Prior MI [61]. (B) ≥2 Prior MIs [61]. (C) Recent MI_<2 yr_ [61]. (D) Recent MI_<1 yr_ [62]. (E) Prior MI + MVD [61]. ARR, absolute risk reduction; CV, cardiovascular; FOURIER, **F**urther Cardiovascular **Ou**tcomes **R**esearch With PCSK9 **I**nhibition in Subjects With **E**levated **R**isk; MI, myocardial infarction; MVD, multivessel disease; NNT, number needed to treat; RRR, relative risk reduction; PCSK9, proprotein convertase subtilisin-kexin type 9.

Recent analyses of large PCSK9i trials have identified subsets of patients with established ASCVD who are at increased risk of CV events and would derive the largest absolute benefit from LLT intensification with PCSK9i therapy [16, 17]. Further to the results in all patients with ASCVD in the FOURIER trial (Fig. 2A) [16], analyses revealed evolocumab treatment reduced the risk of major CV outcomes by 18–30% compared with placebo in subgroups of patients with prior MI with or without residual multivessel disease (Fig. 3) [61, 62], prior percutaneous coronary intervention (PCI; Fig. 2B) [63], and PAD (Fig. 2C) [64], suggesting the absolute benefits of evolocumab are enhanced in these vulnerable patients considering their higher absolute risk of CV events. Likewise, evolocumab was shown to reduce the risk of major CV outcomes compared with placebo in patients with diabetes mellitus (Fig. 2D) [65]. These results correspond to a number needed to treat (NNT) of just 29–50 patients with evolocumab over approximately 3 years, depending on the patient type, in addition to statin therapy and in line with the aforementioned study populations. This and other data on vulnerable patients with ASCVD are described in section 9 on evolocumab safety.

### 5.1 Patients with Acute Coronary Syndrome (ACS) 

Elevated LDL-C is associated with an increased risk of recurrent or adverse CV events in patients with ACS [66]. The impact of evolocumab on patients with ACS was investigated in both the **EVO**locumab for Early Reduction of LDL-Cholesterol Levels in **P**atients With **A**cute **C**oronary **S**yndromes (EVOPACS) and **EV**olocumab in **A**cute **C**oronary **S**yndrome (EVACS) studies (Table 6) [40, 42]. EVOPACS investigated the 8-week feasibility, safety, and LDL-C effects of evolocumab added to statin therapy during the in-hospital phase of ACS, compared with placebo, which also included statin therapy. Most patients (62%) were screened for study participation within <24 hours of patient-reported symptom onset and all within <72 hours [40]. Evolocumab treatment reduced LDL-C levels by 40.7% compared with placebo, with LDL-C reductions observed as early as 4 weeks post-ACS and maintained at 8 weeks. Additionally, 95.7% of patients treated with evolocumab upon their ACS achieved LDL-C <1.8 mmol/L (<70 mg/dL) and 90.1% achieved LDL-C <1.4 mmol/L (<55 mg/dL) compared with 37.6% and 10.7%, respectively, in the placebo group at 8 weeks [40]. LDL-C reductions were consistent regardless of type of ACS [40]. Further, there were no new safety issues with early post-ACS initiation of evolocumab.

The EVACS study included patients with NSTEMI and troponin I ≥5 ng/mL on background statin therapy, though not all were dose-optimized upon clinical presentation [42]. Still, LDL-C reductions from baseline were observed as early as day 1 post-evolocumab initiation, with significant reductions compared with placebo as early as day 3. LDL-C was 0.74 mmol/L (28.6 mg/dL) lower for patients on evolocumab compared with placebo at 30 days, reflecting a calculated 31% reduction. Further, 80.8% of patients on evolocumab achieved LDL-C ≤1.8 mmol/L (≤70 mg/dL) and 65.4% achieved ≤1.4 mmol/L (≤55 mg/dL), compared with only 38.1% and 23.8%, respectively, with placebo.

Altogether, the results from EVOPACS and EVACS demonstrate prompt addition of evolocumab in the hospital after ACS rapidly and significantly reduces LDL-C, compared with statin alone, in patients who most require LDLC reductions to reduce the risk of further CV events. Importantly, most patients were capable of achieving LDL-C levels below guideline-recommended thresholds for patients at increased CV risk prior to hospital discharge.

### 5.2 Patients with Prior MI

Patients with acute MI have higher rates of subsequent major CV events and mortality relative to patients with ASCVD in general [6]. In patients in the FOURIER trial who experienced prior MI, evolocumab demonstrated consistent LDL-C reductions of 59–61% at 48 weeks from baseline, regardless of the time since most recent MI, number of prior MIs, or the presence of residual multivessel CAD, with a median achieved LDL-C of 0.75–0.78 mmol/L (29–30 mg/dL) [61]. Indeed, the proportion of patients who achieved the LDL-C target threshold of <1.0 mmol/L (<40 mg/dL) for patients with recurrent CV events was similar between patients with a recent MI ≤1 year ago (63.8%) and >1 year ago (63.1%) [62]. Further, evolocumab reduced the risk of the composite of CV death, MI, or stroke by 25% in patients with a prior MI compared with placebo, over a median follow-up of only 2.2 years (Fig. 3A). This risk reduction with evolocumab compared with placebo was consistent in patients with ≥2 prior MIs (21%; Fig. 3B), patients with a recent MI ≤2 years ago (24%; Fig. 3C), patients with a recent MI ≤1 year ago (25%; Fig. 3D) and patients with residual multivessel disease (30%; Fig. 3E) [61, 62]. Collectively, these results demonstrate the significant benefit of evolocumab in reducing the residual risk of MACE in patients with prior MI, particularly in those who are at the greatest risk for a subsequent event early in the trajectory of their disease.

### 5.3 Patients with Prior PCI and Impact on Coronary Revascularization

A blinded, post-hoc analysis of the FOURIER trial revealed evolocumab reduced the risk of any coronary revascularization by 22%, simple PCI by 22%, complex PCI by 33%, coronary artery bypass grafting (CABG) surgery by 24%, and complex revascularization (the composite of complex PCI or CABG) by 29% [67]. Hence, these results suggest evolocumab may shift the risk from more complex revascularization procedures towards simple PCI or no revascularization at all. Interestingly, the magnitude of complex revascularization risk reduction with evolocumab tended to increase over time, from 20% in the first year post-evolocumab initiation to 41% beyond the second year. Patients who have undergone PCI are at high residual risk for CV events, including subsequent MI and coronary revascularization [68]. In a prespecified analysis of patients with prior PCI in the FOURIER trial, evolocumab treatment reduced LDL-C by 60.8% at 48 weeks compared with placebo [63]. Furthermore, evolocumab reduced the risk of the composite of CV death, MI, or coronary revascularization by 18% compared with placebo, over a median follow-up of 2.2 years (Fig. 2B), which did not significantly differ based on time since last PCI. Interestingly, CV risk reduction was observed almost immediately after evolocumab initiation, before growing over time, emphasizing the importance of early LDL-C lowering, especially in vulnerable patients, for the opportunity to derive the greatest benefit in the long term.

### 5.4 Patients with PAD

Patients with PAD have an increased risk of major CV events, including CV death, MI, and stroke, compared with patients with stable ASCVD [69, 70]. In patients with PAD in the FOURIER trial, treatment with evolocumab reduced LDL-C by 59% after 48 weeks compared with placebo, with LDL-C levels maintained over time [64]. Furthermore, evolocumab-treated patients had a 27% reduced risk of the composite of CV death, MI, or stroke compared with placebo, over a median follow-up of 2.2 years (Fig. 2C). Additionally, this was the first study to demonstrate a benefit of intensive LDL-C lowering for major adverse limb events (MALE) risk, including the composite of acute limb ischemia, major amputation, or urgent revascularization, with evolocumab treatment in all patients (with or without PAD) conferring a 42% reduced risk compared with placebo. There was a roughly linear relationship between reductions in LDL-C and the risk of MALE, down to an LDL-C of 0.26 mmol/L (10 mg/dL), highlighting the benefits of significant LDL-C lowering in patients with PAD. Further, evolocumab reduced the risk of the combination of MACE and MALE by 49%, yielding an NNT of 16 over 2.5 years for patients with PAD. Finally, there was no impact of evolocumab on any additional treatment emergent adverse events in patients with PAD.

### 5.5 Patients with Diabetes Mellitus

The prevalence of diabetes mellitus has gradually increased globally over the past decades [5], and given the association of diabetes mellitus with increased risk for CV disease morbidity and mortality, there is a need for effective strategies to lower LDL-C in this population [71]. The BANTING study investigated the impact of evolocumab in patients with type 2 diabetes mellitus and hypercholesterolemia or mixed dyslipidemia and on background statin therapy (Table 6) [39]. Evolocumab significantly reduced LDL-C by 54.3% from baseline, compared with 1.1% for placebo at week 12 [39]. Furthermore, 84.5% of patients on evolocumab achieved LDL-C ≤1.8 mmol/L (≤70 mg/dL) and 65.5% achieved an LDL-C reduction of ≥50%, compared with 15.4% and 0.8%, respectively, for placebo [39]. A substantial proportion of patients in the FOURIER trial had diabetes (n = 11,031; 40%) [65], enhancing the CV risk in these patients with ASCVD. In these patients, evolocumab treatment reduced the risk of the composite of CV death, MI, or stroke by 18% compared with placebo (Fig. 2D). The magnitude of CV risk reduction increased over time post-evolocumab initiation, from 13% in the first year to 25% afterwards, emphasizing the importance of early LDL-C lowering in patients with diabetes mellitus, for the opportunity to derive the greatest benefit in the long term. Finally, there was no increase in incident diabetes mellitus in patients who did not have diabetes at baseline, nor did evolocumab worsen glycemic control in patients with diabetes mellitus, as described in section 9 on evolocumab safety.

### 5.6 Patients with Metabolic Syndrome

Metabolic syndrome, comprising three or more of abdominal obesity, hypertension, hyperglycemia, hypertriglyceridemia, and/or low levels of HDL-C, is recognized as a risk factor for major CV events [2, 3, 4, 5]. In patients with metabolic syndrome in the FOURIER trial, compared with placebo, evolocumab reduced LDL-C by 57.7% at 48 weeks as well as the risk of the composite of CV death, MI, or stroke by 24% over a median follow-up of 2.2 years [72]. Hence, these results demonstrate the benefit of evolocumab in reducing CV risk in patients with several risk factors.

## 6. Evolocumab Efficacy in Patients with Statin Intolerance

The reported incidence of statin-associated muscle symptoms in observational studies ranges from 5 to 29% of treated patients, varying by statin and dose [73, 74, 75, 76]; hence, studies have investigated the use of alternative treatments to reduce LDL-C levels and improve CV outcomes in patients with statin intolerance [15, 77]. The impact of evolocumab vs. ezetimibe in patients with statin intolerance due to muscle-related adverse events was investigated in the GAUSS-3 study (Table 6) [38]. For the co-primary end point of mean change in LDL-C for the mean of weeks 22 and 24 (which approximates mean treatment effect), a 54.5% reduction was observed with evolocumab and a 16.7% reduction with ezetimibe. For the other co-primary end point of mean change in LDL-C at week 24 (which reflects effects at the end of the dosing interval), a 52.8% reduction was observed with evolocumab and a 16.7% reduction with ezetimibe. Additionally, the proportion of patients who achieved mean LDL-C <1.8 mmol/L (<70 mg/dL) was 29.9% for evolocumab and 1.4% for ezetimibe for weeks 22/24, and 27.4% and 0%, respectively, at week 24. In addition, both treatments were well tolerated, with muscle symptoms reported in 20.7% of patients on evolocumab and 28.8% patients on ezetimibe. Altogether, these results demonstrate evolocumab is an efficacious and safe monotherapy in patients with statin intolerance, which may provide increased opportunity to achieve LDL-C levels below guideline-recommended thresholds in a population that has historically struggled to do so. Hence, evolocumab is globally indicated for patients with statin intolerance and is recommended as an effective LLT in statin intolerance guidelines [78].

## 7. Efficacy in Special Patient Populations at High-Risk of ASCVD (HeFH, HoHF, HIV)

### 7.1 Patients with FH

FH is an autosomal dominant genetic disorder characterized by chronically elevated circulating LDL-C, which can accelerate the development of ASCVD, with an estimated 10- to 20-fold increased risk compared with normolipidemic individuals [79, 80, 81]. Patients with HeFH commonly experience LDL-C levels >4.9 mmol/L (>190 mg/dL), and HoFH is even more severe with LDL-C levels >13 mmol/L (>503 mg/dL) [82]. Clinical trials have demonstrated evolocumab added to statin therapy reduces LDL-C compared with placebo (+statin) in both adult and pediatric patients with either HeFH or HoFH [37, 41, 83]. The phase III **R**ed**U**ction of LDL-C With PCSK9 Inhibi**T**ion in **HE**te**R**ozygous **F**amilial Hyperch**O**leste**R**olemia **D**isorder Study-2 (RUTHERFORD-2) trial is the largest reported global trial of patients with HeFH treated with a PCSK9i (Table 6) [37]. Both doses of evolocumab (i.e., 140 mg Q2W or 420 mg QM) reduced LDL-C by ~60% compared with placebo at week 12, with reductions observed as early as week 2 and remaining consistent up to week 12 [37]. Furthermore, LDL-C <1.8 mmol/L (≤70 mg/dL) was achieved by ≥63% of patients receiving either dose of evolocumab, compared with 2% in each of the placebo groups [37].

Evolocumab efficacy in HeFH was also investigated in pediatric patients (aged 10–17 years) in the phase III Trial Assessing Efficacy, Safety and Tolerability of PCSK9 In**H**ibition in Pedi**A**tric S**U**bject**S **With Gen**E**tic LDL Disorde**R**s (HAUSER) study (Table 6) [41]. Evolocumab reduced LDL-C by 38.3% compared with placebo at week 24 [41]. Furthermore, 74% of pediatric patients achieved LDL-C <3.4 mmol/L (<130 mg/dL), the target LDL-C threshold within the study, and 45% achieved an LDL-C reduction of ≥50% [41].

In the open-label, single-arm TAUSSIG study (Table 6), evolocumab reduced LDL-C by 1.94 ± 3.22 mmol/L (74.9 ± 124.5 mg/dL) in patients with HoFH and by 2.33 ± 1.60 mmol/L (90.6 ± 61.9 mg/dL) in patients with severe HeFH after 4.1 years, corresponding to 24.0% and 47.2% reductions, respectively [83]. The individual variability in evolocumab response in patients with HoFH is attributed to differences in *LDLR* expression resulting from their disease, with the non-responders lacking functioning or available LDL receptors [84]. Furthermore, although the magnitude of LDL-C reduction may be less in homozygous or compound heterozygous double LDLR mutation carriers, the LDL-C reduction is sustained [83]. Evolocumab efficacy has also been demonstrated in patients with a rare subtype of HeFH due to PCSK9 gain-of-function mutations [83]. Additionally, a CV event rate of 2.7% per year was reported [83], which was markedly lower than expected given the high risk of CV events in patients with HoFH and HeFH reported in other studies [85]. Moreover, of the 61 patients undergoing apheresis at enrollment, 9% with HoFH and 48% with HeFH were able to discontinue apheresis, sparing on average 36 months of apheresis treatment over the course of the study [83]. Of the 26% of patients who stopped apheresis altogether, 62.5% were able to do so within 90 days of starting evolocumab treatment [83].

In summary, FH is a serious incurable disease that substantially increases the risk of primary as well as secondary ASCVD, even in pediatric patients. These results demonstrate the addition of evolocumab to background statin therapy confers clinically significant LDL-C reductions to achieve levels below guideline-recommended thresholds, in a population wherein statins alone are often insufficient. Further, no additional evolocumab safety issues were identified in these studies, as described in section 9 on evolocumab safety. Hence, evolocumab is globally indicated in patients with HeFH and HoFH and is guideline-recommended to ultimately reduce the substantial CV risk associated with lifelong exposure to elevated LDL-C [2, 3, 4, 5].

### 7.2 Patients with HIV 

Patients with HIV are considered to be at high risk for ASCVD because of traditional risk factors such as dyslipidemia, insulin resistance, tobacco, and hypertension, and also due to the immune-mediated changes associated with HIV [86, 87, 88]. Moreover, evidence suggests certain antiretroviral therapies (ARTs) may increase the risk of ASCVD, especially first-generation protease inhibitors that can result in endothelial dysfunction or metabolic imbalances [86, 88, 89, 90]. Considering the minimal LLT studies in this high-risk patient population, the impact of LLT is not well-understood. In the phase III BEIJERINCK study (Table 6), evolocumab treatment reduced LDL-C by 56.9% at week 24 compared with placebo in patients with HIV receiving maximally tolerated statin therapy [43]. Additionally, 73.3% and 72.5% of patients on evolocumab achieved LDL-C <1.8 mmol/L (<70 mg/dL) and a ≥50% reduction from baseline, compared with 7.9% and 0.7% of patients, respectively, with placebo. Lastly, no neutralizing antibodies were developed against evolocumab, and no safety issues were identified, as described in section 9 on evolocumab safety. Thus, these results demonstrate evolocumab is an efficacious add-on to background statin therapy to achieve LDL-C levels below guideline-recommended thresholds in patients with HIV, an immunocompromised population with complex etiology underlying their increased risk of ASCVD.

## 8. Real-World Evidence of Evolocumab Effectiveness

Real-world studies have assured the safety and benefits of LLT intensification with PSCK9i to reduce LDL-C as observed in clinical trials are reproducible in routine practice. In the U.S. **G**etting to an Impr**O**ved **U**nderstanding of Low-Density **L**ipoprotein Cholesterol and **D**yslipidemia Management: A Registry of High Cardiovascular Risk Subjects in the United States (GOULD) study (N = 5006) in patients with established ASCVD and LDL-C above goal, 52.4% of patients on a PCSK9i for 2 years achieved LDL-C <1.8 mmol/L (<70 mg/dL) compared with ≤33.9% of other patients not on a PCSK9i at baseline, though some of whom may have later been initiated on a PCSK9i during the 2-year follow-up [22]. Further, 39.9% of patients on PCSK9i achieved LDL-C <1.4 mmol/L (<55 mg/dL), compared with ≤11.9% of other patients. In a Canadian real-world study of patients with acute MI (N = 15,283), patients on a PCSK9i with background LLT achieved the greatest LDL-C reductions, to a median of 0.9 mmol/L (35 mg/dL) [21]. This reflected a 66.5% LDL-C reduction from baseline compared with a 27.8% reduction in patients on high-intensity statin therapy alone. Further, a greater proportion of patients initiated on a PCSK9i with background LLT achieved guideline-recommended LDL-C thresholds compared with all other LLT regimens, with 77.7% and 75.0% more achieving LDL-C <1.8 and <1.4 mmol/L (<70 and <55 mg/dL) post-LLT intensification, respectively, compared with 32.4% and 24.9% initiated on high-intensity statin therapy alone. Importantly, 57.1% of patients achieved the intensive LDL-C goal of <1.0 mmol/L (<40 mg/dL) for the most vulnerable patients, compared with only 9.6% patients initiated on high-intensity statin therapy alone. Likewise, the following real-world studies of evolocumab specifically have also confirmed its effectiveness in reducing LDL-C levels in heterogeneous patient populations at high and very high CV risk, with and without established ASCVD, and across several countries and continents.

### 8.1 HEYMANS Study

In the HEYMANS study (Table 7) of patients initiated on evolocumab as part of routine clinical care across 12 European countries (N = 1951), 41% of patients were not on background statin and/or ezetimibe therapy at evolocumab initiation and 60% had reported statin intolerance [44]. Consistent with the FOURIER trial and OLE [16, 36], evolocumab therapy was associated with a 58% reduction in LDL-C from baseline after 3 months, which was maintained over 30 months of follow-up, all despite the heterogenous patient population on varying background LLT. Approximately 60% of patients achieved ≥50% LDL-C reduction throughout the study. Further, 56% of patients at high CV risk achieved the guideline-recommended LDL-C goal of <1.8 mmol/L (<70 mg/dL) and 60% of patients at very high CV risk achieved the more aggressive goal of <1.4 mmol/L (<55 mg/dL). Overall, a greater proportion of patients achieved below LDL-C thresholds on evolocumab plus background LLT compared with those on evolocumab monotherapy, consistent with the science showing the impact of PCSK9 inhibition on LDL-C clearance is enhanced when used in combination with a statin [91].

### 8.2 ZERBINI Study

**Fig. 4.**
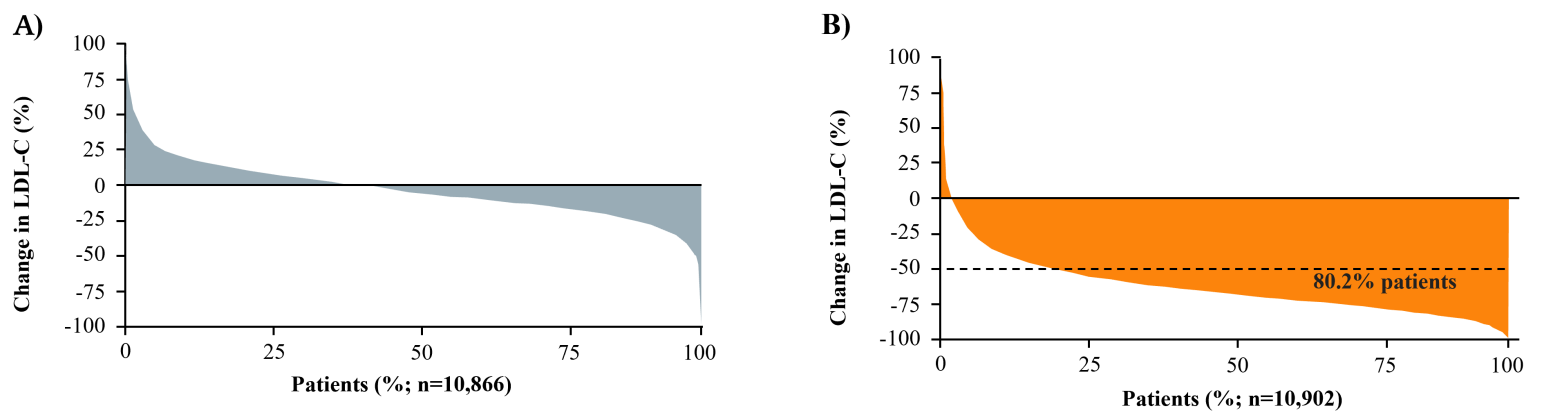
**Waterfall plots of the distribution of percentage change in LDL-C from baseline**. (A) Week 4 in Placebo Group of FOURIER. (B) Week 4 in Evolocumab Group of ${\mathrm{FOURIER}}^{1,2}$. ^1^In the FOURIER trial, at 1-year post-evolocumab initiation, 94.7% of patients achieved a ≥50% LDL-C reduction; ^2^Data represent patients with an LDL-C measure at baseline (measured within 6 months prior to initiation of evolocumab) and their minimum LDL-C measure during the 12-month study follow-up period. (Figure permission obtained from [57]). FOURIER, **F**urther Cardiovascular **Ou**tcomes **R**esearch With PCSK9 **I**nhibition in Subjects With **E**levated **R**isk; LDL-C, low-density lipoprotein cholesterol; PCSK9, proprotein convertase subtilisin-kexin type 9.

In the ZERBINI study of patients initiated on evolocumab as part of routine clinical care in three continents and five countries (Table 7), including Canada, Mexico, Colombia, Saudi Arabia, and Kuwait (N = 578), 15% of patients were not on background statin and/or ezetimibe therapy at evolocumab initiation and 36% had reported statin intolerance (ranging 7.7–61.8% across the different countries) [46]. In the full heterogeneous cohort on varying background LLT, evolocumab therapy was associated with a 70% reduction in LDL-C from baseline, which was maintained over a 12-month period. Further, 76.0% of patients achieved ≥50% reduction in LDL-C, which is consistent with the FOURIER trial wherein 80% of patients achieved ≥50% LDL-C reduction at 4 weeks (Fig. 4, Ref. [57]), and 94.7% at 12 months [16]. In the ZERBINI study, guideline-recommended LDL-C thresholds of <1.8, <1.4, and <1.0 mmol/L (<70, <55, and <40 mg/dL) were achieved by 75%, 64%, and 47% of patients, respectively [46]. Notably, these LDL-C outcomes were consistent across high- and very high-risk patients, including those with ASCVD or FH, ASCVD with FH, ≥2 ASCVD conditions, and ASCVD with diabetes, with >50% of very high-risk patients achieving the most intensive LDL-C goal of <1.0 mmol/L (<40 mg/dL) recommended for patients who experienced a second CV event within the previous 2 years [3]. These real-word LDL-C reductions associated with evolocumab therapy in vulnerable patients are consistent with those from sub-analyses of the FOURIER trial [65]. Finally, all ZERBINI study outcomes were consistent across sub-analyses of the Canadian [92], Colombian [93], and Middle Eastern [94] cohorts.

## 9. Evolocumab Safety Across PROFICIO Program of Clinical Trials and RWE Studies

**Fig. 5.**
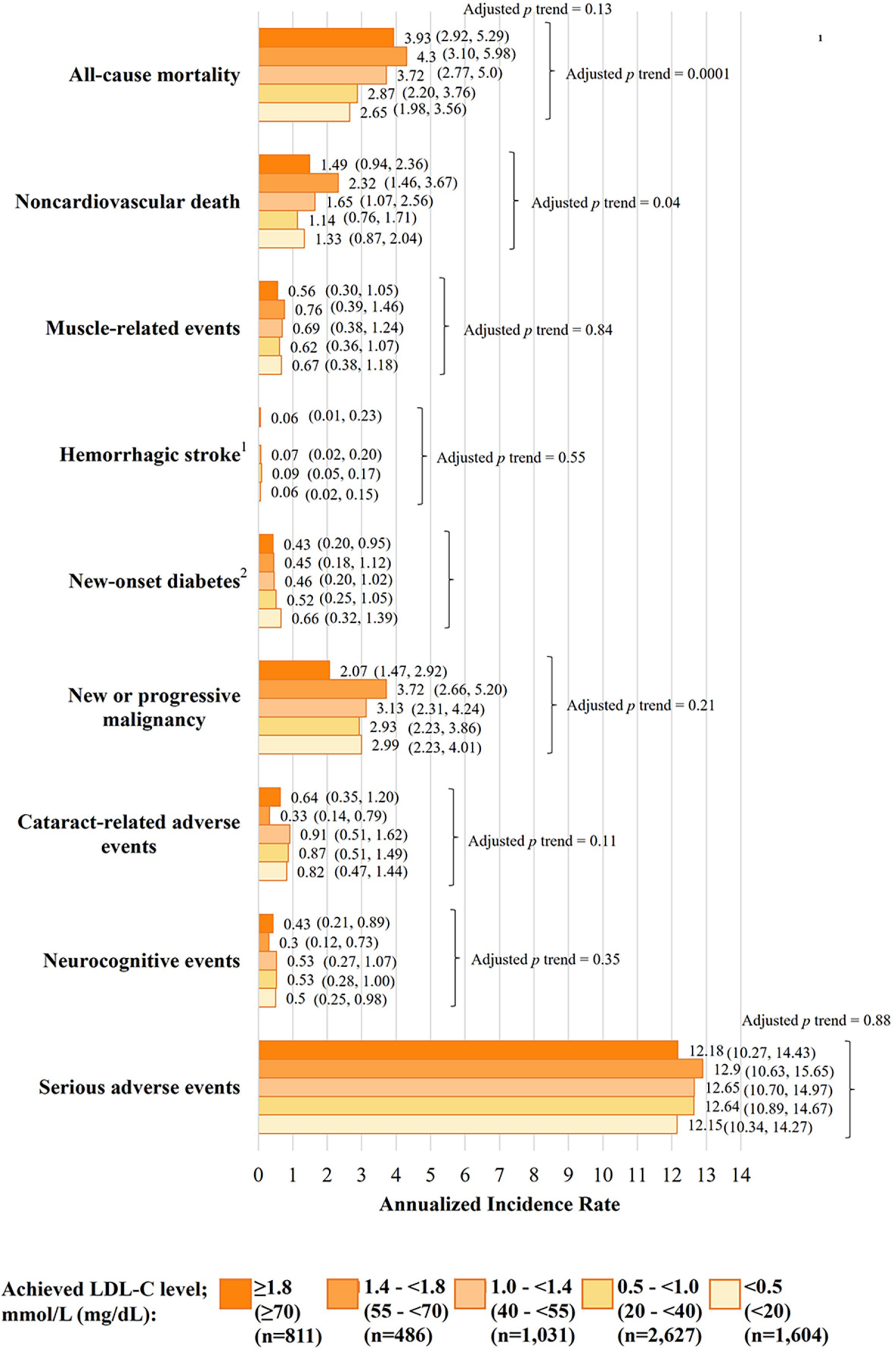
**Safety outcomes according to achieved LDL-C in the FOURIER-OLE over a median evolocumab exposure of 5.0 years**. Data are annualized incidence rates (95% CIs) and have been adjusted for age, body mass index, sex, race (White vs. other), previous myocardial infarction, nonhemorrhagic stroke, history of PAD, history of diabetes, current smoking, high statin use, ezetimibe use, and lipoprotein(a) at 12 weeks. ^1^Unadjusted data are presented because of small numbers of event rates. ^2^Additional adjustments were made for baseline hemoglobin A1c level for this endpoint and the denominator excludes patients diagnosed with diabetes before or at enrollment into FOURIER-OLE. CI, confidence interval; FOURIER-OLE, **F**urther Cardiovascular **Ou**tcomes **R**esearch With PCSK9 **I**nhibition in Subjects With **E**levated **R**isk **O**pen-**L**abel **E**xtension; LDL-C, low-density lipoprotein-cholesterol; PAD, peripheral arterial disease; PCSK9, proprotein convertase subtilisin-kexin type 9.

Evolocumab has consistently been shown to have a favourable safety profile across the PROFICIO program of clinical trials and RWE studies in patients at high and very high CV risk [16, 36]. In the FOURIER trial, there were no differences between the evolocumab and placebo groups in the rates of adverse events, serious adverse events, or treatment emergent adverse events that lead to study discontinuation, with the exception of injection site reactions, which were more common with evolocumab compared with placebo (2.1% vs. 1.6%, respectively) [16]. Likewise, there was no increase in any additional adverse events during the 8.4 years of follow-up in the FOURIER-OLE, the longest study of PCSK9 inhibition in ASCVD to date [36]. Importantly, long-term evolocumab safety was consistent across all levels of achieved LDL-C, down to <0.5 mmol/L (<20 mg/dL) (Fig. 5) [60]. With the FOURIER-OLE being the first LLT study to investigate the effects of these very low achieved LDL-C values, this novel finding advances the current understanding of significant and sustained LDL-C lowering and of the effect of LLT on the regulation of circulating lipoproteins in general.

Evolocumab safety has also been shown to be consistent in clinical trials of special populations of patients at risk of or with established ASCVD. As mentioned, in the FOURIER trial, evolocumab did not increase the risk of new-onset diabetes mellitus or worsen glycemic control in patients with diabetes mellitus [65], and in the BEIJERINCK study, evolocumab safety was confirmed in immunocompromised patients with HIV [43]. Further, in the EBBINGHAUS trial in a subset of 1204 patients from the FOURIER trial, there was no significant difference in cognitive function between patients who received evolocumab or placebo over a median of 19.4 months in the context of the very low levels of achieved LDL-C [95]. Moreover, in pediatric patients with HeFH aged 10–17 years in the HAUSER study, evolocumab safety was consistent with that reported in trials of adult patients, and did not affect measures of pubertal development, growth variables, or carotid intima-media thickness after 24 weeks [41]. Likewise, after 80 weeks in the HAUSER-OLE, adverse event rates were consistent with the trial phase and none led to evolocumab discontinuation [96]. Ultimately, these evolocumab safety data met the rigorous criteria for international approval for use in pediatric patients (with HeFH and HoFH) [20]. Importantly, evolocumab has been confirmed to have no negative impact on cognition in both pediatric and adult patients. In fact, in the HAUSER study in pediatric patients with HeFH, abnormal and clinically important cognitive decline occurred less frequently in the evolocumab vs. placebo group [97].

The ZERBINI RWE study and sub-analyses by country confirmed these clinical data, with only 3.3% of patients reporting an adverse event, yet none of a serious nature [46, 92, 93, 94]. Notably, only 1 puncture site ecchymosis was reported in the ZERBINI study (0.2% of patients), which may be reflective of improved patient counselling on self-injection and administration skills over time. Further, the low incidence of myalgia (0.5%) in the ZERBINI study is also reassuring, especially considering 35.6% of patients had reported statin intolerance, suggesting evolocumab does not exacerbate muscle symptoms in susceptible patients [46]. Altogether, these results suggest a favourable safety profile for evolocumab, with the only reported contraindication being patients who are hypersensitive to evolocumab [20].

## 10. Evolocumab Persistence

Consistent with robust evolocumab effectiveness and tolerability, there is a growing body of RWE demonstrating a high persistence rate (>90%) for evolocumab [22, 44, 46]. In the U.S. GOULD study and European HEYMANS study, 93% of patients were still taking their mAb PCSK9i at 2 years and 1 year, respectively [22, 45]. Likewise, in the international ZERBINI study, 90.2% of patients persisted on evolocumab over 12 months [46], which was consistent in the Canadian [92], Colombian [93], and Middle Eastern [94] sub-analyses. Underlying evolocumab persistence may be its ease and convenience of use, with many patients able to self-administer it at home without necessitating frequent clinic visits, as shown in the THOMAS studies [33]. Interestingly, most patients in the FOURIER trial (90%) [16] as well as in the ZERBINI real-world study (98.8%) [46] chose the Q2W vs. QM evolocumab dosing regimen. Nevertheless, a separate analysis of the **O**pen Label **S**tudy of **L**ong-T**ER**m Evaluation Against LDL-C Trial (OSLER)-2 study showed <10% of patients within each regimen switched their dosing regimen during an average of 11 months of follow-up [98]. These data are important considering a lack of persistence to CV therapy is associated with poor clinical outcomes, including hospitalization and mortality, especially in high-risk patients. International RWE shows a lack of persistence to statin therapy, even among patients following a CV event [99, 100, 101], which may be attributed to intolerance and fear of known side effects [102, 103]. Hence, these results suggest evolocumab provides patients with the opportunity to remain on guideline-recommended LLT as prescribed, to achieve significant, sustained, long-term LDL-C reductions in order to gain the most CV benefit.

## 11. Ongoing Evolocumab Studies

Evolocumab evidence generation continues to advance, with an ongoing commitment to understand the potential efficacy benefits and safety of evolocumab in unstudied patient populations with increased CV risk. A summary of select major ongoing studies at the time of this review is presented here:

### 11.1 VESALIUS-CV

Effect of E**V**olocumab in Pati**E**nt**S** at High C**A**rdiovascu**L**ar R**I**sk Witho**U**t Prior Myocardial Infarction or **S**troke (VESALIUS)-CV is a phase III multinational trial assessing the effect of optimized LLT intensification with evolocumab in reducing first major CV events in adults with established ASCVD or diabetes mellitus, without prior MI or stroke, compared with placebo (+optimized LLT) [104]. Inclusion criteria require elevated LDL-C and presence of at least one of the following high-risk conditions at screening: significant CAD, atherosclerotic cerebrovascular disease, PAD, and/or diabetes mellitus. Primary outcomes are time to coronary heart disease death, MI, or ischemic stroke or any ischemia-driven arterial stroke over a minimum of 4.5 years of follow-up. This study will be the first CV outcome study with a PCSK9i to include a large cohort of primary prevention patients.

### 11.2 EVOLVE-MI

The phase IV A Pragmatic, Randomized, Multicenter Trial of **EVOL**ocumab Administered **V**ery **E**arly to Reduce the Risk of Cardiovascular Events in Patients Hospitalized With Acute **M**yocardial **I**nfarction (EVOLVE-MI) open-label trial is evaluating the effect of early treatment with evolocumab plus routine LLT vs. routine LLT alone to reduce MI, ischemic stroke, arterial revascularization, and all-cause mortality in adult patients hospitalized for an acute MI (NSTEMI and STEMI) [105]. This is a pragmatic study to assess the impact of evolocumab initiated within 10 days of the index MI in the acute setting compared with standard of care. The primary outcome is the total (first and subsequent) composite of MI, ischemic stroke, any arterial revascularization procedure, and all-cause mortality over approximately 3.5 years of follow-up.

### 11.3 NEWTON-CABG

NEWTON-CABG is a phase IV trial evaluating the effect of evolocumab added to routine statin therapy on vein graft patency after CABG surgery compared with placebo (+statin) [106]. The primary outcome is saphenous vein graft disease rate (VGDR) 24 months post-CABG, with VGDR defined as the proportion of vein grafts with significant stenosis or total occlusion (≥50%) on 64-slice (or greater) cardiac computed tomography angiography (CTA) or clinically indicated coronary angiography.

Taken together, the results of these novel studies will advance the current understanding of the impact of LLT intensification with evolocumab on major CV outcomes in vulnerable patients. Further, these studies will address a data gap and may help shape clinical practice for certain patient types for whom current guidelines are unclear, such as those with diabetes mellitus without established ASCVD.

## 12. Conclusions

This review provides evidence for the significant clinical and real-world CV benefits of PCSK9 inhibition with the mAb evolocumab in patients with and without established ASCVD. The various evolocumab data summarized from the 50 clinical trials and RWE studies in >51,000 patients to date over the past 13 years consistently showed significant reductions in LDL-C (Tables 2,3,4,5,6,7), with most patients achieving LDL-C levels well below international guideline-recommended thresholds to reduce the risk of initial and recurrent CV events. Indeed, the efficacy of evolocumab to reduce the risk of MACE has been shown down to the lowest LDL-C levels ever studied (<0.5 mmol/L; <20 mg/dL), with the results emphasizing the importance of early, intensive, and continued LDL-C reductions, especially in vulnerable patients with ASCVD, for the opportunity to derive the greatest benefit in the long term. Further, evolocumab has been shown to have a favourable safety profile across all achieved LDL-C levels and in various patient types, including pediatric patients with FH and immunocompromised patients with HIV, with no increase in adverse events in the real-world setting or long-term, apart from local injection site reactions. Nonetheless, the use of evolocumab should always take into consideration the benefit: risk profile for individual patients. Evolocumab efficacy and safety data are supported by a high persistence rate (>90%) reported in RWE studies, which may also be attributable to simple and convenient at-home self-administration. In conclusion, the wealth of evolocumab data reviewed herein, together with anecdotal clinical experience since its global approval 8 years ago, collectively from >2.5 million patients to date, have advanced our understanding of the vital importance of significant LDL-C reduction in ASCVD and demonstrate the potential of evolocumab to address a current healthcare gap in a variety of high- and very high-risk patients.
