# Erratum: Leiter *et al*. Review of Evolocumab for the Reduction of LDL Cholesterol and Secondary Prevention of Atherosclerotic Cardiovascular Disease. Reviews in Cardiovascular Medicine. 2024; 25: 190

**DOI:** 10.31083/j.rcm2510383

**Published:** 2024-10-24

**Authors:** Lawrence A. Leiter, Robert A. Hegele, Vivien Brown, Jean Bergeron, Erin S. Mackinnon, G. B. John Mancini

**Affiliations:** ^1^Li Ka Shing Knowledge Institute, St. Michael’s Hospital, University of Toronto, Toronto, ON M5S 1A8, Canada; ^2^Departments of Medicine and Biochemistry, Schulich School of Medicine and Dentistry, Western University, London, ON N6A 5C1, Canada; ^3^Department of Family and Community Medicine, Temerty Faculty of Medicine, University of Toronto, Toronto, ON M5G 1V7, Canada; ^4^Departments of Laboratory Medicine and Medicine, Centre Hospitalier Universitaire de Québec, Université Laval, Québec, QC G1V 4G2, Canada; ^5^Amgen Canada Inc., ON L5N 0A4, Canada; ^6^Centre for Cardiovascular Innovation, Division of Cardiology, University of British Columbia, Vancouver, BC V5Z 1M9, Canada

The authors wish to make the following corrections to this paper [1]:

1. On the right side of page 3, the text in line 17, the sentence “at a willingness-to-pay threshold of $100,00 CAD per additional QALY gained. Furthermore, for every 100 patients treated for lifetime, the addition of evolocumab to optimized LLT was estimated to prevent ~52 CV events, of which 7 would be fatal.” should be changed to “at a willingness-to-pay threshold of $100,000 CAD per additional QALY gained [23]. Furthermore, for every 100 patients treated for lifetime, the addition of evolocumab to optimized LLT was estimated to prevent ~52 CV events, of which 7 would be fatal [23].”

2. Adjust the formatting instructions for Fig. 5, and move the “adjusted *p* trend = 0.13” at the top of the figure to the back of the curly bracket of “New-onset diabetes” in line 5.

**Figure:**
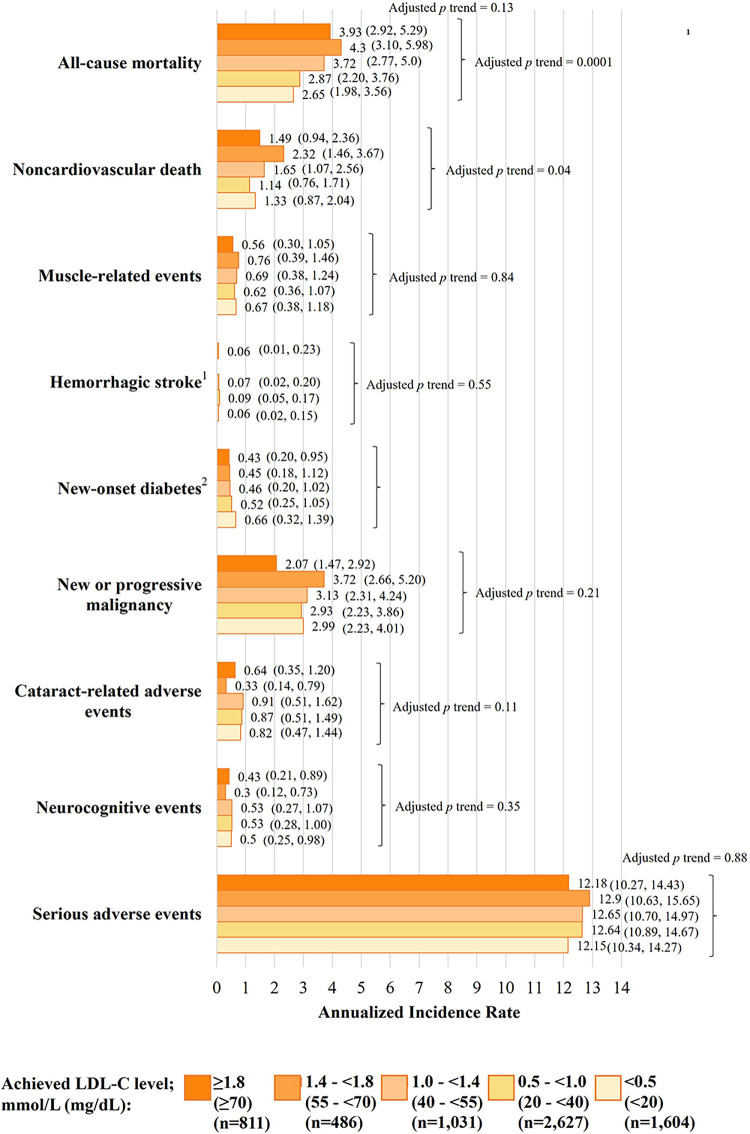
Error Fig. 5.

**Figure:**
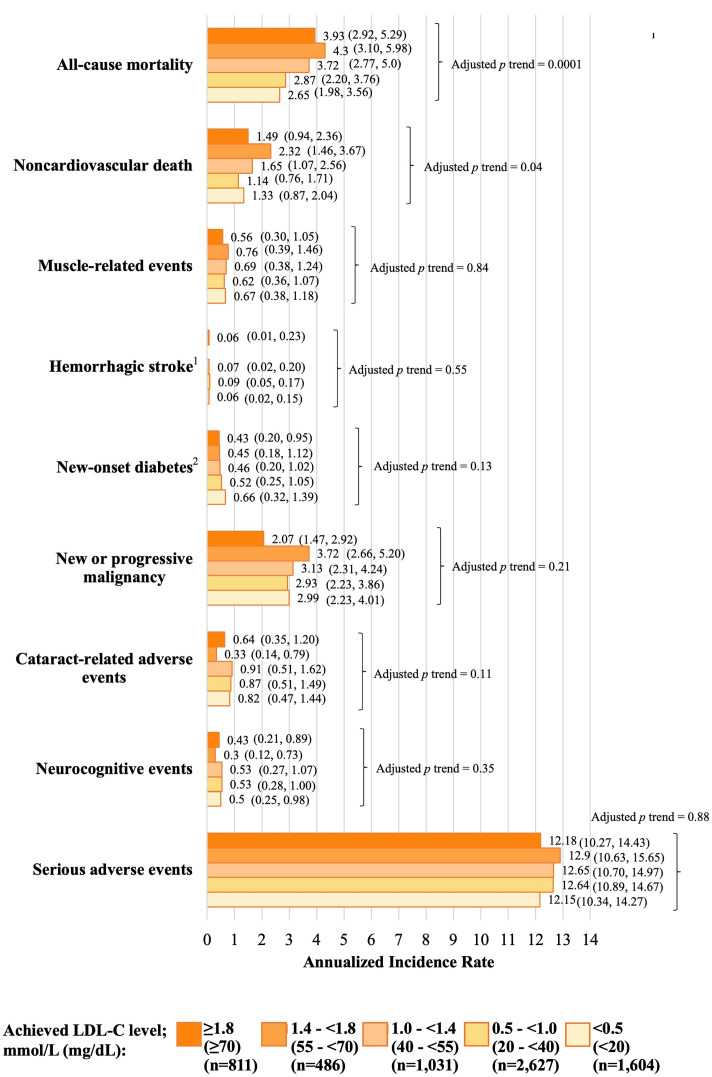
**Fig. 5. Safety outcomes according to achieved LDL-C in the FOURIER-OLE over a median evolocumab exposure of 5.0 years**. Data are annualized incidence rates (95% CIs) and have been adjusted for age, body mass index, sex, race (White vs. other), previous myocardial infarction, nonhemorrhagic stroke, history of PAD, history of diabetes, current smoking, high statin use, ezetimibe use, and lipoprotein(a) at 12 weeks. ^1^Unadjusted data are presented because of small numbers of event rates. ^2^Additional adjustments were made for baseline hemoglobin A1c level for this endpoint and the denominator excludes patients diagnosed with diabetes before or at enrollment into FOURIER-OLE. CI, confidence interval; FOURIER-OLE, **F**urther Cardiovascular **Ou**tcomes **R**esearch With PCSK9 **I**nhibition in Subjects With **E**levated **R**isk **O**pen-**L**abel **E**xtension; LDL-C, low-density lipoprotein-cholesterol; PAD, peripheral arterial disease; PCSK9, proprotein convertase subtilisin-kexin type 9.

This has been corrected as of August 26, 2024. The authors apologize for these errors.
